# Unusually high mechanical stability of bacterial adhesin extender domains having calcium clamps

**DOI:** 10.1371/journal.pone.0174682

**Published:** 2017-04-04

**Authors:** Anneloes S. Oude Vrielink, Tyler D. R. Vance, Arthur M. de Jong, Peter L. Davies, Ilja K. Voets

**Affiliations:** 1 Institute for Complex Molecular Systems, Eindhoven University of Technology, Eindhoven, Netherlands; 2 Laboratory of Macromolecular and Organic Chemistry, Department of Chemical Engineering and Chemistry, Eindhoven University of Technology, Eindhoven, Netherlands; 3 Department of Biomedical and Molecular Sciences, Queen’s University, Kingston, Canada; 4 Laboratory of Molecular Biosensing for Medical Diagnostics, Department of Applied Physics, Eindhoven University of Technology, Eindhoven, Netherlands; 5 Laboratory of Physical Chemistry, Department of Chemical Engineering and Chemistry, Eindhoven University of Technology, Eindhoven, Netherlands; Semmelweis Egyetem, HUNGARY

## Abstract

To gain insight into the relationship between protein structure and mechanical stability, single molecule force spectroscopy experiments on proteins with diverse structure and topology are needed. Here, we measured the mechanical stability of extender domains of two bacterial adhesins *Mp*AFP and *Mh*Lap, in an atomic force microscope. We find that both proteins are remarkably stable to pulling forces between their N- and C- terminal ends. At a pulling speed of 1 μm/s, the *Mp*AFP extender domain fails at an unfolding force *F*_u_ = 348 ± 37 pN and *Mh*Lap at *F*_u_ = 306 ± 51 pN in buffer with 10 mM Ca^2+^. These forces place both extender domains well above the mechanical stability of many other β-sandwich domains in mechanostable proteins. We propose that the increased stability of *Mp*AFP and *Mh*Lap is due to a combination of both hydrogen bonding between parallel terminal strands and intra-molecular coordination of calcium ions.

## Introduction

Single molecule force spectroscopy (SMFS) has been used to measure the mechanical stability of proteins and to elucidate their underlying molecular mechanisms. [1, 2] Tens of proteins have been subjected to force spectroscopy measurements, which have revealed that β-sheet proteins tend to be more stable, relative to α-helical proteins. [3, 4] Several of the strongest natural proteins reported to date have β-sandwich folds, and are often repetitive domains of larger, extracellular proteins, where high mechanical strength is an asset for proper function in the face of environmental pressures. For example, the Ig-like domains 27 and 32 of the intracellular human muscle protein titin unfold at roughly 200 pN and 300 pN, respectively (400 nm/s pulling speed) [5, 6], while cohesin I modules from the extracellular *Clostridium* scaffoldins, CipA and CipC connecting region, unfold at forces above 400 pN (400 nm/s pulling speed). [7] A key determinant of the high mechanical stability of these proteins is the presence of hydrogen bonds between parallel β-strands found at the termini of each repeat, which form the mechanical clamp: a structural region in a protein that is responsible for the enhanced resistance to stretching. [8] The cohesin I modules from CipA and CipC (known as c7A and c1C, respectively) have two mechanical clamps in tandem, which would explain their substantially higher mechanical stability relative to the titin repeats which only contain a single clamp. The resistance to shearing by parallel, terminal β-strands has also been shown to enhance the mechanical stability in non-mechanical proteins, such as protein L and GB1. [9, 10] Furthermore, this motif has been used to single out proteins with high mechanical stability in the protein data bank (PDB). [11] Coarse-grained molecular dynamics simulations with protein structures deposited in the PDB, have led to the classification of several types of mechanical clamp motifs including shear (of which there are parallel-, antiparallel-, disconnected-, and supported-), delocalized, zipper and torsion clamp motifs. [12–14] Apart from proteins with cysteine slipknots, the scaffoldins c1C and c7A were among the strongest proteins, with a high predicted mechanical stability that could be experimentally verified. [7]

Besides hydrogen bonding, there are many other factors that influence the mechanical stability of proteins, such as core packing, solvation, entropic effects and ligand binding. [15] *Cao et al*. showed that the stability of protein GB1 could be enhanced by as much as 100 pN by incorporating a bi-histidine site that can reversibly bind Ni^2+^ ions. Furthermore, it was shown that the increase in stability depends on the positioning of this Ni^2+^-chelation site inside the protein. [16] Calcium binding was shown to enhance the mechanical stability of the proteins M-crystallin and gelsolin. [17, 18] The unfolding force of M-crystallin increased from *F*_u_ = 91 ± 21 pN to *F*_u_ = 125 ± 20 pN upon binding two calcium ions. [17] The unfolding force of gelsolin gradually increased with calcium concentration, from *F*_u_ = 23.9 ± 6.1 pN in the absence of calcium to *F*_u_ = 41.0 ± 6.1 pN in 50 μM calcium for the holo-protein which binds one calcium ion. [18]

**Fig 1:**
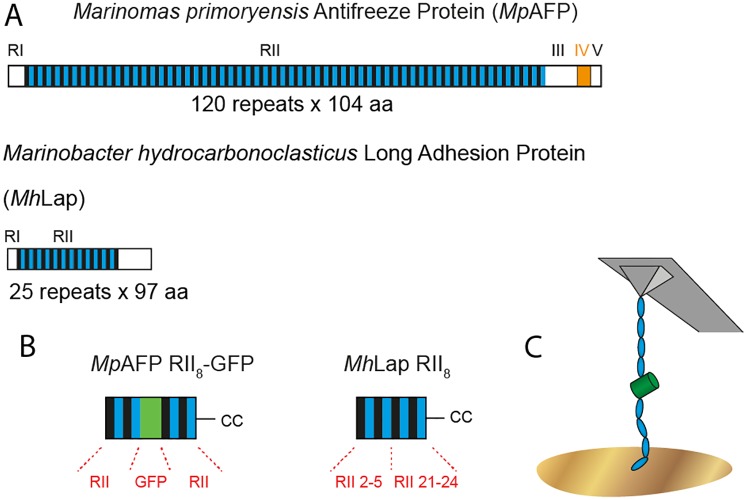
Schematic representation of full adhesins *Mp*AFP and *Mh*Lap and corresponding SMFS constructs. The mechanical stability of region II of the full adhesin *Mp*AFP (1.5 MDa) and *Mh*Lap (0.3 MDa) (A) is investigated using octameric constructs (B). Region II of *Mp*AFP consists of 120 identical 104-amino-acid repeats, whereas region II of *Mh*Lap consists of 25 repeats of 97 amino acids, with on average 76% sequence identity between subsequent repeats. *Mp*AFP RII_8_-GFP consists of eight *Mp*AFP RII repeats separated into two sections of tetra-tandemers by a GFP protein included in the middle which serves as internal force calibration standard. *Mh*Lap RII_8_ consists of repeats 2–5 and 21–24. Both constructs have two C-terminal cysteines to promote the interaction of the proteins with the gold surface. The full amino acid sequences of the protein constructs are given in Section A in S1 Supporting Information. The pickup of the adhesin construct *Mp*AFP RII_8_-GFP by the AFM tip is shown schematically in (C).

Here we investigate the mechanical strength of two Ca^2+^-dependent extender domains from bacterial adhesins by AFM single molecule force spectroscopy. The Antarctic marine bacterium *Marinomonas primoryensis* adheres to ice on brackish lakes via the ice-binding adhesin *Mp*AFP. This mechanism may ensure access to the higher levels of oxygen and nutrients found in the upper strata of its habitat. [19–21] *Marinobacter hydrocarbonoclasticus* is a marine bacterium originally isolated along the French Mediterranean coast, where it forms biofilms on hydrophobic organic compounds which it degrades for use as a source of carbon and energy. [22, 23] The mechanisms behind the formation of these oleolytic biofilms have yet to be elucidated, but the *M*. *hydrocarbonoclasticus* genome does contain an adhesin, dubbed *Mh*Lap, which is proposed to play a critical role. [24] Both *Mp*AFP and *Mh*Lap have similar domain architectures, with the N- and C-terminal regions being separated by a large stretch of a repetitive sequence, known as region II (RII). RII comprises 90% of the mass of *Mp*AFP and consists of 120 identical 104-amino acid repeats of an immunoglobulin-like β-sandwich, which fold in a Ca^2+^-dependent manner. [20, 25] Recent small angle X-ray scattering measurements on a RII tetra-tandemer demonstrate that calcium also rigidifies the RII domain of *Mp*AFP. [20] RII of *Mh*Lap comprises 25 repeats of a 97-amino-acid domain with on average 76% sequence identity between repeats at the protein level (Fig 1). For both adhesins, region II forms the link between the bacterium and its natural substrate, and as such is predicted to require high stability and strength to combat environmental shear forces.

We find that the extender domains of region II of both *Mp*AFP and *Mh*Lap have remarkably high stability in the presence of 10 mM calcium, with *F*_u_ = 348 ± 37 pN and *F*_u_ = 306 ± 51 pN, respectively, at a pulling speed of 1 μm/s. In addition, these high stabilities are found to be calcium dependent, decreasing dramatically with decreased Ca^2+^ concentration.

## Results and discussion

### High mechanical stability of *Mp*AFP and *Mh*Lap in the presence of 10 mM Ca^2+^

**Fig 2:**
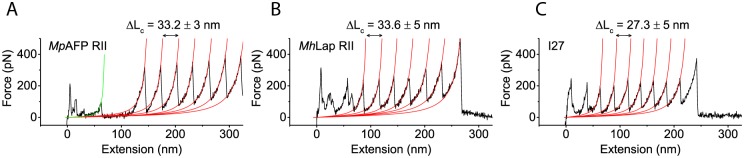
Typical sawtooth-like force-extension curves. Force curves of (A) *Mp*AFP RII_8_-GFP, (B) *Mh*Lap RII_8_ and (C) I27^RS^_8_ were obtained at a pulling speed of 1 μm/s and 10 mM Ca^2+^. The worm-like chain (WLC) model was applied to analyze the observed unfolding peaks from which we obtain values for a contour length increase Δ*L*_c_ upon unfolding and a persistence length *L*_p_. The force-distance curve of the *Mp*AFP RII_8_-GFP displays seven peaks corresponding to the unfolding of RII monomers (red WLC fit) and a much smaller peak at a small extension corresponding to GFP unfolding (green WLC fit). At a 1 μm/s pulling speed we obtained Δ*L*_c_ = 33.2 ± 3 nm, Δ*L*_c_ = 33.6 ± 5 nm and Δ*L*_c_ = 27.3 ± 5 nm for *Mp*AFP RII, *Mh*Lap RII and I27, respectively. Experimental data are shown in black; red and green solid lines correspond to WLC fits.

To assess the mechanical stability of region II of the Ca^2+^-dependent bacterial adhesins *Mp*AFP from *Marinomonas primoryensis* and *Mh*Lap from *Marinobacter hydrocarbonoclasticus*, single molecule force spectroscopy experiments were performed on two representative linear octameric constructs: *Mp*AFP RII_8_-GFP and *Mh*Lap RII_8_ (Fig 1) at 10 mM Ca^2+^, and on an exemplary strong protein, I27^RS^_8_. Unfolding curves obtained by stretching the octa-tandemers *Mp*AFP RII_8_-GFP (Fig 2A), *Mh*Lap RII_8_ (Fig 2B) and I27^RS^_8_ (Fig 2C) at a pulling speed of 1 μm/s displayed sawtooth-like patterns with several well-defined peaks of comparable peak force and length, characteristic for the stretching of polyproteins composed of identical or nearly identical subunits that give way at similar unfolding forces and extensions.

We used the worm-like chain (WLC) model (Eq 5) to analyze the unfolding peaks in these force-extension curves in terms of a contour length increase Δ*L*_*c*_ and a persistence length *L*_*p*_. For *Mp*AFP RII at a pulling speed of 1 μm/s (Fig 2A), we derived Δ*L*_*c*_ = 33.2 ± 2.8 nm (average ± s.d., N = 277) and *L*_*p*_ = 0.3 ± 0.1 nm (N = 479). Similarly, we obtained Δ*L*_*c*_ = 33.6 ± 5 nm (N = 687) and *L*_*p*_ = 0.2 ± 0.1 (N = 945) for *Mh*Lap RII. To compare these experimental values with literature values, we compute the contour length increase per amino acid *L*_*aa*_ from the total number of amino acids per monomer, *N*_*aa*_, and the total length of the unfolded monomer, *L*, which is given by the contour length increase Δ*L*_*c*_ upon unfolding and the N- to C- terminal distance *d*_N;C_ of the folded monomer as follows:

$$L_{aa}=\frac{\Delta L_{c}+d_{\text{N};\text{C}}}{N_{aa}}$$ (1)

The crystal structure of the *Mp*AFP RII tetra-tandemer (PDB code 4p99) provided *d*_N;C_ = 4.8 nm and *N*_*aa*_ = 104, which gave a contour length increase per amino acid *L*_*aa*_ = 38.0 / 104 = 0.37 nm/aa, which is in good agreement with literature values. [26, 27] Because the crystal structure of *Mh*Lap is unknown, the Protein Homology/analogy recognition Engine (Phyre2) was used to predict a protein structure for the amino acid sequence of *Mh*Lap RII based on homology modelling. [28] From this homology model we derived *d*_N;C_ = 3.38, which yielded *L*_*aa*_ = 0.38 nm/aa; i.e., again agreeing well with literature values. Interestingly, the extender domain structure of *Mp*AFP RII was the template that provided the greatest confidence for the structure prediction of *Mh*Lap RII repeats 2–5 and 21–24, with each repeat sharing approximately 30% sequence identity at the protein level. This indicates that the proteins are homologous and are likely to have the same overall fold (see Section C in S1 Supporting Information for further details).

Next we determined the unfolding forces *F*_u_ from the height of the protein unfolding peak minus the height of the baseline. At a pulling speed of 1 μm/s, *Mp*AFP RII unfolded at *F*_u_ = 348 ± 45 pN (N = 518), while *Mh*Lap RII unfolded at *F*_u_ = 306 ± 51 pN (N = 1006). This indicates that both adhesins have an unusually high mechanical stability. Only few natural proteins are known to unfold at higher forces, including the cohesins c7A and c1C, which unfolded at *F*_u_ = 480 ± 14 pN and *F*_u_ = 425 ± 9 pN, respectively, at a pulling speed of 400 nm/s [7] and the isopeptide bond-delimited loop of Gram-positive bacterial pili CnaA domains SpaA and FimA which unfold at even higher forces of 525 ± 65 pN and 690 ± 70 pN respectively. [29] The high stability of bacterial adhesins *Mp*AFP RII and *Mh*Lap RII are in line with reported nN magnitude adhesion forces between microbes and substrates. [30, 31]

To validate the SMFS results, we also measured the unfolding force and contour length increase of two well-characterized proteins: GFP and I27. [5, 26, 32, 33] We first probed the unfolding of GFP, which was included in the middle of the *Mp*AFP RII_8_-GFP construct. In chimeric polyproteins, the weakest domain of the protein chain unfolds first. [3] Since there are four RII repeats on each side of GFP, an unfolding of more than four RII repeats indicates that GFP has been subjected to a mechanical force of more than 300 pN. The mechanical stability of GFP is only 104 ± 40 pN (300 nm/s), [26] therefore we can assume that in those cases, GFP is unfolded as well. However, a GFP unfolding peak was observed before the first *Mp*AFP RII peak in only ~15% of force-extension curves with five or more *Mp*AFP RII peaks (see Section G in S1 Supporting Information). An estimated value of the unfolding force *F*_u_ = 88 ± 7 pN and unfolding length Δ*L* = 74.4 ± 3.9 nm of GFP was obtained from four force curves. The unfolding length of GFP is 2.3 times longer than that of the RII extender domains, which is in excellent agreement with the 2.3 times longer amino acid chain length of GFP (239 aa) compared to the *Mp*AFP RII extender domain (104 aa). The obtained unfolding force of GFP is somewhat lower than the unfolding force *F*_u_ = 104 ± 40 pN (300 nm/s) reported by Dietz and Rief. but within the standard deviation. [26] In other force curves with five or more *Mp*AFP RII peaks, GFP unfolding peaks were absent (~50%), or masked by nonspecific interactions between the AFM tip and substrate (~35%, see Section G in S1 Supporting Information for example curves). We speculate that inclusion of GFP between consecutive *Mp*AFP RII tetra-tandemers might hinder folding of GFP into its native conformation. We therefore decided to also perform pulling experiments on the mechanostable octa-tandemeric titin construct I27^RS^_8_ (Fig 2C). These yielded *F*_u_ = 218 ± 29 pN (N = 349) and Δ*L* = 25.4 ± 3 nm (N = 347), which are comparable to literature values (*F*_u_ = 204 ± 26 pN and Δ*L* = 24.1 ± 0.34 nm). [5] The unfolding length Δ*L* is the distance between consecutive I27 peak positions. Hence, we have validated our SMFS results and conclude that the two Ca^2+^-dependent bacterial adhesins have a high mechanical stability in the presence of 10 mM Ca^2+^ when stretched between terminal ends.

Since repeats 2–5 and 21–24 in *Mh*Lap RII_8_ are highly similar (on average 78% sequence identity) yet not identical, we studied whether this variation in amino acid sequence translates into a variation in mechanical stability. To this end, we determined the relative standard deviation of unfolding forces $c_{v}\;=\;\;\frac{\sigma }{\mu }$ in force curves with five or more RII unfolding peaks. Here, *σ* is the standard deviation of unfolding forces and μ is the average unfolding force of a particular force curve. On average, $\bar{c_{v}}\;=\;14.3\;\pm \;4.8\%$ (N = 65 curves) for *Mh*Lap RII, which is only slightly higher than the standard deviation in force curves of *Mp*AFP RII $\bar{c_{v}}\;=\;8.0\;\pm \;2.9\%$ (N = 12 curves) and I27 $\bar{c_{v}}\;=\;10.1\;\pm \;3.3\%$ (N = 18 curves) at the same pulling speed. Apparently, the variation in amino acid sequence of *Mh*Lap repeats 2–5 and 21–24 does not have a large effect on the unfolding force, which is consistent with the close resemblance in the folds of *Mh*Lap RII repeats predicted by Phyre2.

**Fig 3:**
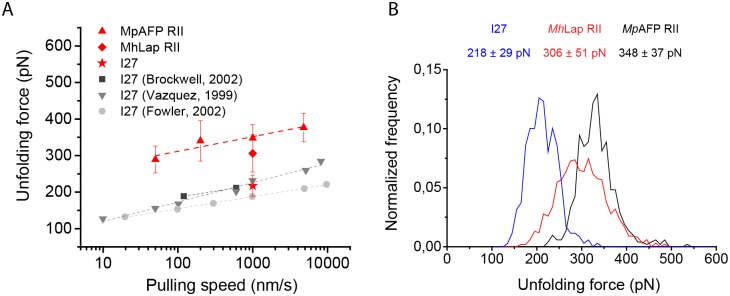
Pulling speed dependence of *Mp*AFP RII and unfolding force histograms of *Mp*AFP RII, *Mh*Lap RII and I27. (A) A linear dependence of *Mp*AFP RII unfolding force with pulling speed is visible. The measured unfolding forces for I27 at 1 μm/s pulling speed were in agreement with data of I27 taken from Brockwell *et al*. [32], Carrion-Vazquez *et al*. [5] and Fowler *et al*. [33]. (B) Normalized histograms of measured unfolding forces of I27 (N = 349), *Mh*Lap RII (N = 1006) and *Mp*AFP RII (N = 518) at 1 μm/s pulling speed.

To further assess the mechanical stability of *Mp*AFP RII, we measured the loading rate dependence of the unfolding force by performing pulling experiments on *Mp*AFP RII at loading rates of 50 nm/s, 200 nm/s, 1 μm/s and 4.88 μm/s (Fig 3). *Mp*AFP RII unfolded at higher forces than I27 for all pulling speeds, and *F*_u_ increased with increasing pulling speed.

Bell’s model describes how the unfolding rate constant of a protein depends on the pulling force

$$k_{u}\;\left(F\right)=\nu \kappa \cdot e^{\frac{-\Delta G-Fx_{u}}{k_{B}T}}=k_{u}^{0}\cdot e^{\frac{Fx_{u}}{k_{B}T}}$$ (2)

where *k*_*u*_ is the rate constant at force *F*, $k_{u}^{0}$ the rate constant at zero force, *v* is the vibrational frequency at the transition state, *k* the transmission coefficient, Δ*G* the activation energy for unfolding, *x*_*u*_ the distance to the transition state, *k*_*B*_ the Boltzmann constant and *T* the temperature. [34, 35] At higher pulling speeds protein unfolding is observed at higher forces, and *x*_*u*_ and $k_{u}^{0}$ can be determined from the slope and intercept of a plot of the unfolding force *F* versus the loading rate *k*_*c*_*v* [36]

$$F\;\left(k_{c}v\right)=\frac{k_{B}T}{x_{u}}\text{ln}\left(\frac{k_{c}vx_{u}}{k_{B}Tk_{u}^{0}}\right).$$ (3)

Hoffmann *et al*. showed that mechanically stable proteins do not deform much before reaching the transition state, resulting in a small value for *x*_*u*_. [4] From a linear fit of Eq 3, we estimated *x*_*u*_ ~ 0.2 nm and $k_{u}^{0}\;\sim \;0.003$ s^-1^ for *Mp*AFP RII (Figure C in S1 Supporting Information). As expected for a mechanically stable protein, the distance to the transition state *x*_*u*_ ~ 0.2 nm of *Mp*AFP RII is small, in comparison with values reported for other proteins 0.14 ≤ *x*_*u*_ ≤ 2 nm. [4]

### Calcium enhances the mechanical stability of *Mp*AFP RII

Circular dichroism (CD) spectroscopy experiments on *Mp*AFP RII extender domains showed that the protein is unstructured in the absence of calcium, but gradually folds into a β-stranded structure as the calcium concentration increases. [25] Several bound calcium ions are visible in the crystal structure of *Mp*AFP RII tetra-tandemers at high Ca^2+^ concentrations. Three Ca^2+^ ions reside within each *Mp*AFP RII monomer, while one Ca^2+^ ion is bound between monomer repeats. [20] Furthermore, the thermal stability of RII repeats is affected by calcium (see Figure H in S1 Supporting Information). Since *Mp*AFP RII monomers fold in a Ca^2+^-dependent manner, we investigated the impact of calcium on the mechanical stability of the protein.

**Fig 4:**
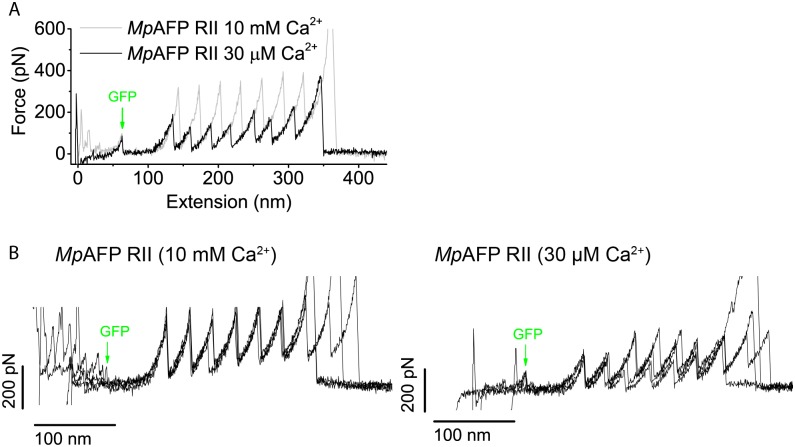
*Mp*AFP RII unfolding force depends on Ca^2+^ concentration. (A) Overlay of force curves of *Mp*AFP RII_8_-GFP at 10 mM and 30 μM Ca^2+^. Force peaks of *Mp*AFP RII are on average ~100 pN lower when the protein is in 30 μM calcium compared to the force peaks of *Mp*AFP RII in 10 mM calcium. Force peak of GFP is unaffected, which is as expected since no Ca^2+^ ions are bound to GFP. (B) Overlay of four force curves of *Mp*AFP RII_8_-GFP in 10 mM Ca^2+^ (left) and 30 μM Ca^2+^ (right). A larger spread in unfolding force peaks is observed for *Mp*AFP RII at 30 μM Ca^2+^ compared to *Mp*AFP RII in 10 mM Ca^2+^. All force measurements were performed at 1 μm/s pulling speed.

To investigate whether calcium enhances the mechanical stability of *Mp*AFP RII, we performed SMFS measurements at low (30 μM) and high (10 mM) free calcium concentrations corresponding to a partially (30 μM) and completely (10mM) folded state of the RII monomer according to CD spectroscopy. [25] Samples were prepared by dialyzing *Mp*AFP RII solution to buffer with 0.1 mM EDTA, after which Ca^2+^ was added to the solution to a free Ca^2+^ ion concentration of either 30 μM or 10 mM (see Materials and methods). Based on CD measurements using comparable dialysis methods, RII monomers are predominantly unfolded at low Ca^2+^ concentration. [25] Therefore we assume that all structurally-relevant Ca^2+^ ions are removed from the protein when dialyzing to buffer containing 0.1 mM EDTA. Fig 4A shows an overlay of two typical force-distance curves obtained for *Mp*AFP RII_8_^-^GFP at low (solid black line) and high (solid grey line) Ca^2+^ concentration. Unmistakably, the unfolding peaks are lower at a free calcium concentration of 30 μM than at 10 mM Ca^2+^. On average, ~100 pN lower unfolding forces were observed for *Mp*AFP RII in buffer with 30 μM calcium (*F*_u_ = 245 ± 58 pN, N = 180) compared to *Mp*AFP RII in buffer with 10 mM calcium (*F*_u_ = 347 ± 37 pN, N = 518) (see Section B in S1 Supporting Information). Hence, the partially unfolded conformation of an *Mp*AFP RII extender domain at low calcium concentrations is mechanically less stable than the completely folded conformation attained at 10 mM Ca^2+^. By contrast, the non-calcium binding protein I27 shows no significant difference in mechanical stability when measured either with 10 mM calcium or without calcium (see Figure E in S1 Supporting Information). This is in agreement with the findings of D. J. Echelman *et al*. who show that an unrelated non-calcium binding protein FimA showed no gain in stability upon the addition of 10 mM Ca^2+^. [29]

Fig 4B displays an overlay of several force-distance curves for *Mp*AFP RII_8_-GFP at low and high calcium concentrations. Interestingly, the variation in unfolding forces is larger at 30 μM Ca^2+^ than at 10 mM Ca^2+^. To analyze this in a more quantitative fashion, we determined the relative standard deviation in the force peaks $c_{v}=\;\frac{\sigma }{\mu }$ of each force curve wherein five or more *Mp*AFP RII unfolding peaks were observed. We find $\bar{c_{v}}\;=\;25.9\;\pm \;9.7\%$ (N = 4) for *Mp*AFP RII at 30 μM Ca^2+^, which is large compared to $\bar{c_{v}}\;=\;8.0\;\pm \;2.9\%$ (N = 12) for *Mp*AFP RII at 10 mM Ca^2+^ and $\bar{c_{v}}\;=\;10.1\;\pm \;3.3\%$ (N = 18) for I27. It seems plausible to attribute this large variability in mechanostability among different monomers within a single polyprotein chain to differences in the number of bound Ca^2+^ ions in the partially unfolded state at 30 μM Ca^2+^. Since *Mp*AFP RII extender domains have four highly conserved Ca^2+^ binding sites, individual monomers might have between zero and four Ca^2+^ ions bound, which presumably would affect their resistance against unfolding.

Finally, we investigated whether temporary calcium depletion reduces the mechanical stability of *Mp*AFP RII. To this end we compared force-distance curves of *Mp*AFP RII constructs from samples kept throughout in a buffer at 10 mM Ca^2+^ (Figs 2 and 3) to samples subjected to dialysis against 0.1 mM EDTA followed by Ca^2+^ addition (see Materials and methods section for details) to reach the same final 10 mM Ca^2+^ concentration (Fig 4B). We find *F*_u_ = 367 ± 54 pN (N = 105) for dialyzed samples, which is in close agreement with *F*_u_ = 348 ± 37 pN (N = 518) for samples kept at 10 mM Ca^2+^ throughout (i.e., the dialysis step was omitted). Apparently, the transient depletion of calcium has no discernable impact on the mechanical stability of *Mp*AFP RII at 10 mM Ca^2+^, indicating that folding of the monomer into the stable Ca^2+^-bound structure is reversible.

### The high mechanical stability of *Mp*AFP and *Mh*Lap is due to a combination of hydrogen-bonded parallel terminal strands and calcium-mediated clamps

**Fig 5:**
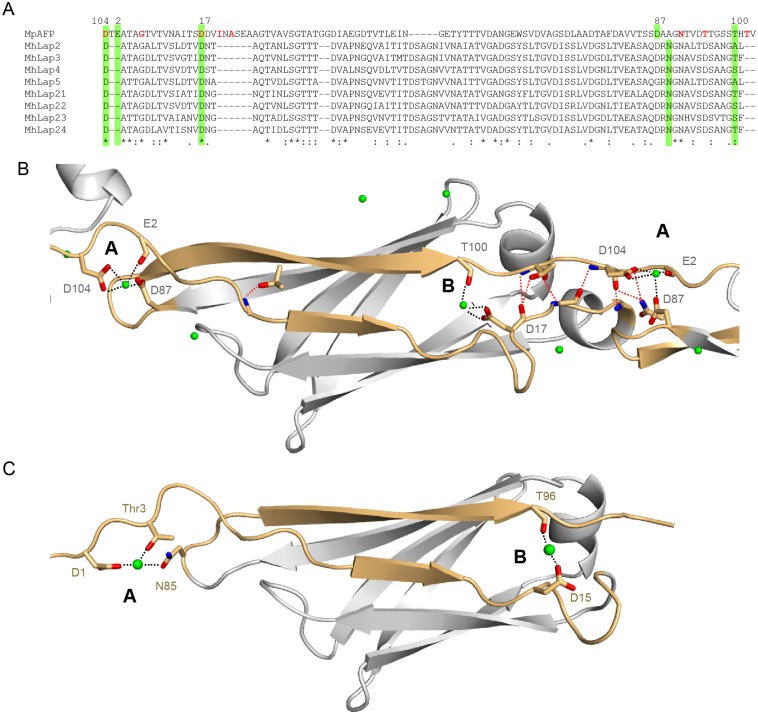
Coordinated calcium ions clamp both *Mp*AFP RII and *Mh*Lap RII extender domains. (A) Amino acid sequence alignment of the *Mp*AFP RII extender domain with eight *Mh*Lap RII repeats used in the octa-tandemer construct. Residues highlighted in green coordinate the calcium ions A and B that are involved in clamping. Residues involved in hydrogen bonding of terminal strands are indicated in red. (B) Crystal structure of the third repeat of *Mp*AFP RII tetra-tandemer (4P99) and (C) Phyre2 model of *Mh*Lap RII repeat 3. Calcium ions involved in clamping are labelled A and B. Interactions of *Mp*AFP RII residues with Ca^2+^ ions are indicated with green dashed lines. Hydrogen bonds are indicated with red dashed lines (see Section D in S1 Supporting Information for details).

As mentioned above, the majority of the most stable natural proteins measured to date possess hydrogen-bonded terminal parallel β-strands, including cohesins c7A (*F*_u_ = 480 ± 14 pN, *v* = 400 nm/s), [7] c1C (*F*_u_ = 425 ± 9 pN, *v* = 400 nm/s), [7] c2A (*F*_u_ = 214 ± 8 pN, *v* = 400 nm/s), [7] ubiquitin (*F*_u_ = 230 ± 34 pN, *v* = 1 μm/s) [37], I32 (*F*_u_ = 298 ± 24 pN, *v* = 400 nm/s) and I27 from titin (*F*_u_ = 204 ± 26 pN, *v* = 400 nm/s). [38] *Mp*AFP RII contains hydrogen bonds between terminal strands, as is evident from the crystal structure of the tetra-tandemer (4P99) (Fig 5B). Some of these hydrogen bonds are located in between subsequent repeats (see Table H in S1 Supporting Information). Based on the Phyre2 model, there are also hydrogen bonds between terminal strands of *Mh*Lap RII (see Table J in S1 Supporting Information).

Interestingly, both *Mp*AFP RII and *Mh*Lap RII are more stable than many of the other proteins listed above even though they are strengthened by the same motif. This raises the question what then is responsible for the increased mechanostability of the bacterial adhesins. Fig 4 demonstrates the reliance of *Mp*AFP RII on calcium for stability: its unfolding force is reduced by ~100 pN upon a reduction in the calcium concentration from 10 mM to 30 μM, which indicates that apart from hydrogen bonding, the coordination of calcium is an important contribution to the mechanical stability. The crystal structure of the tetra-tandemer of *Mp*AFP RII shows the location of four conserved calcium ions per repeat, several of which connect secondary structures. The calcium ions labeled as A and B anchor terminal strands or nearby portions of the protein (Fig 5B, see Table I in S1 Supporting Information). Most residues that coordinate these calciums in *Mp*AFP RII are also conserved in *Mh*Lap RII repeats (Fig 5A). This strongly suggests maintenance of these ion-binding sites, and allows us to place hypothetical calcium ions in both the N and C-terminal clamp positions of the Phyre2 model (Fig 5C). In conclusion, the coordination of calcium between terminal strands indicates a role in organizing and anchoring the termini in a similar manner to the classical shear mechanical clamp motif. These calcium clamps allow the *Mp*AFP RII and *Mh*Lap RII domains to resist higher forces than hydrogen bonding between their parallel terminal strands could alone.

The high mechanical stability of *Mp*AFP RII and *Mh*Lap RII is in line with the high resistance to unfolding that has been observed in several other bacterial adhesins, indicating that these proteins are optimized for resistance to shearing forces. [29, 39] Specifically, some Gram-positive pili are mechanically inextensible or partly inextensible by the strategic location of isopeptide bonds. [29, 40–43] The extensible part between isopeptide bonds in SpaA and FimA is the most stable natural protein sequence measured to date. In SpaA, binding of a Ca^2+^ ion promoted rapid refolding of the protein into the mechanically most stable state, even though the Ca^2+^ binding site is not located in the clamp region of this protein. [29]

## Conclusions

AFM single molecule force spectroscopy experiments were performed at 10 mM free Ca^2+^ concentrations to determine the mechanical strength of the extender domains (region II) of two Ca^2+^-dependent bacterial adhesins: *Mp*AFP and *Mh*Lap. We find that RII of both bacterial adhesins has an exceptionally high mechanical stability at 10 mM Ca^2+^ with unfolding forces *F*_u_ = 348 ± 37 pN and *F*_u_ = 306 ± 51 pN respectively at a pulling speed of 1 μm/s. The stability of *Mp*AFP RII is drastically reduced at 30 μM Ca^2+^ where the protein is only partially folded. While these proteins are not as strong as the cohesion I modules of CipA and CipC, they still have higher unfolding forces than many single-mechanical clamp proteins like titin I27. The impressive strength of *Mp*AFP RII and *Mh*Lap RII is attributed to a combination of a classical mechanical clamp region and complementary calcium clamps that anchor the terminal strands via coordination to neighboring secondary structure elements. Together, these molecular attributes allow the adhesins' host bacteria to retain advantageous positions in their environments, in the face of strong shear forces.

## Materials and methods

### Materials

Gold substrates were obtained from Ssens (product 1-04-02-000, Glass Disc, 1 inch, 200 nm thick gold layer) and cleaned with piranha solution, which is composed of a 3:1 mixture of sulfuric acid (95%) and hydrogen peroxide (30%). After incubation in piranha solution for 5 min at room temperature, the substrates were rinsed with MilliQ water and stored until use. After AFM experiments, gold substrates were piranha-cleaned before reuse. Atomic force microscopy silicon nitride cantilevers (MLCT-E) with a spring constant *k* ~ 0.1 N/m were purchased from Bruker and used as received.

### Protein engineering

Three protein constructs *Mp*AFP RII_8_-GFP, *Mh*Lap RII_8_ and I27^RS^_8_ were designed and expressed for single molecule force spectroscopy experiments. All three constructs contain two C-terminal cysteines to attach the polyprotein chain covalently to the gold substrate.

#### *Mp*AFP RII_8_-GFP

We designed an *Mp*AFP RII_8_-GFP construct consisting of eight identical *Mp*AFP RII monomers separated in the middle by GFP (see Section A in S1 Supporting Information for amino acid sequences). The construct was inserted into the pET28a expression vector between *Nde*1 and *Xho*1 cut sites, thereby adding an N-terminal Histidine tag. The DNA encoding GFP was inserted using an added *Hind*III cut site. Recombinant plasmid was electroporated into *Escherichia coli* BL21(DE3) and expressed through IPTG induction (1 mM). Following overnight incubation, recombinant protein was extracted from the cells through sonication in the presence of 10 mM CaCl_2_, and purified using Ni^2+^ affinity chromatography. Elution fractions were subjected to size-exclusion chromatography using a Superdex S200 16/60 column (GE Healthcare). *Mp*AFP RII_8_-GFP eluted as a single peak. Fractions were pooled and dialyzed to 50 mM Tris-HCl (pH 9), 200 mM NaCl, 10 mM CaCl_2_, 3 mM TCEP. Dialyzed proteins were stored at -80°C until use.

#### *Mh*Lap RII_8_

The *Mh*Lap RII_8_ octa-tandemer protein consists of repeats 2–5 and 21–24 of RII of *Mh*Lap, with on average 78% sequence identity. It was cloned and expressed using the same protocol as for the *Mp*AFP RII_8_-GFP construct, but without the internal GFP DNA.

#### I27^RS^_8_

To have a basis for comparison, SMFS was also performed on the exemplary strong polyprotein I27^RS^_8_, which is an octa-tandemer of the immunoglobulin-like module 27 of the I band of human cardiac titin. The gene I27^RS^_8_ was constructed according to work of Carrion-Vazquez *et al*, [5] synthesized and cloned into pET15b by GenScript. In this construct, amino acids RS are placed between the I27 repeats (see Section A in S1 Supporting Information for amino acid sequences). The protein was expressed in *Escherichia coli* NiCo21 (DE3) cells (New England Biolabs), using IPTG (0.3 mM) in auto-induction medium. [44] After overnight incubation, protein extraction was initiated through homogenization, and purification of the His-tagged protein was performed by affinity chromatography on Ni-NTA resin, followed by size-exclusion chromatography using Superdex S200 16/600 column (GE Healthcare). The I27^RS^_8_ protein was dialyzed into PBS buffer (137 mM NaCl, 2.7 mM KCl, 10 mM Na_2_HPO_4_, 1.8 mM KH_2_PO_4_, pH 7.4) and stored at -80°C until use. The analysis of I27 in this manuscript are based on the force measurements performed in PBS buffer. To check whether calcium has an influence on the mechanical stability of I27 this protein was dialyzed to 50 mM Tris-HCl (pH 9), 200 mM NaCl, 10 mM CaCl_2_. These control measurements are shown in Figure E in S1 Supporting Information.

### Sample preparation

Samples for SMFS experiments were prepared by incubation of a 300 μl droplet of 50–100 μg/ml protein solution on cleaned gold substrates for 10 min, followed by careful rinsing of the gold substrate with buffer. Experiments on *Mp*AFP RII_8_-GFP and *Mh*Lap RII_8_ were carried out in 50 mM Tris-HCl (pH 9), 200 mM NaCl, 10 mM CaCl_2_, 3 mM TCEP. Experiments on I27^RS^_8_ were carried out in PBS with 3 mM DTT. The reducing agents TCEP or DTT were added fresh to the buffer to effectively reduce the two C-terminal cysteines of the protein constructs.

To determine the influence of Ca^2+^ on the mechanical stability of *Mp*AFP RII_8_-GFP, a 1.45 mg/ml solution of the protein was dialyzed into 50 mM Tris-HCl (pH 9), 200 mM NaCl, 0.1 mM EDTA to remove Ca^2+^, using Spectra/Por regenerated cellulose dialysis membranes, and stored at 4°C. Dialysis was performed overnight and buffer was refreshed twice. Before an SMFS experiment, the protein solution was diluted to 50–100 μg/ml, and brought to a final Ca^2+^ concentration of 0.13 mM or 10.1 mM Ca^2+^ by addition of a small volume from a 5 mM or 250 mM Ca^2+^ stock, respectively. Because of the presence of 0.1 mM EDTA this yields final free Ca^2+^ concentrations of 30 μM or 10 mM. [45]

### Single molecule force spectroscopy measurements and data analysis

Single molecule AFM measurements were carried out on a Bruker BioScope Catalyst atomic force microscope using MLCT-E silicon nitride cantilevers (Bruker). Before each experiment, the cantilever was calibrated in water on bare gold using the ‘thermal tune’ method of the Nanoscope 8.1 R3sr7 software. With this method the deflection of the cantilever is recorded over a 30-s time interval, and Fourier transformed to obtain the power spectral density in the frequency domain. The resonant peak is fitted by

$$A\left(v\right)=A_{0}+A_{DC}\cdot \frac{v_{0}^{2}}{\sqrt{\left(v_{0}^{2}-v^{2}\right)+\frac{v_{0}^{2}v^{2}}{Q^{2}}}}$$ (4)

where *A*(*v*) is the amplitude at frequency *v*, *A*_0_ is the baseline amplitude, *A*_*DC*_ is the amplitude at zero frequency, *v*_0_ is the center frequency of the resonance peak and *Q* is the quality factor. The area under the resonant peak equals the power *P* and is used to derive the cantilever spring constant *k* by $k=\frac{k_{B}T}{\langle z^{2}\rangle }=\frac{k_{B}T}{P}$. In this equation *k*_*B*_ is the Boltzmann constant, *T* is the temperature and 〈*z*^2^〉 is the mean square displacement of the cantilever. The average value of the spring constant determined from three independent calibration measurements was used for further analysis. We obtained spring constants of 0.12 ≤ *k* ≤ 0.22 N/m with an accuracy of 5–10%.

A typical SMFS experiment in an AFM microscope consists of two thousand ramps of 1 μm with 2V deflection and 1-s rest time on the surface. In this manner, force-distance curves were obtained at four different pulling speeds of 50 nm/s, 200 nm/s, 1 μm/s and 4.88 μm/s. Since 1–5% of the 2000 ramps per experiment displayed characteristic sawtooth-like patterns, force-distance curves were filtered in an automated procedure using Nanoscope Analysis 1.5 software. For each pulling speed, at least 120 protein unfolding peaks were analyzed, which were obtained from at least three different experiments using different cantilevers. Analysis of the protein unfolding peaks was performed using PUNIAS software. [46]

Unfolding forces were determined as the height of the protein unfolding peak minus the height of the baseline. The unfolding length Δ*L* was determined from the difference in extension between subsequent peaks. The contour length increase Δ*L*_*c*_ (Fig 2), and persistence length *L*_*p*_ were determined from fits of the worm-like chain (WLC) model to protein unfolding peaks,

$$F\left(x\right)=\left(\frac{k_{B}T}{Lp}\right)\left[\frac{1}{4{\left(1-\frac{x}{L_{c}}\right)}^{2}}-\frac{1}{4}+\frac{x}{L_{c}}\right].$$ (5)

where *F*(*x*) is the force in N, *L*_*c*_ is the contour length of the stretched protein in m, *L*_*p*_ is the persistence length in m, *k*_*B*_ is the Boltzmann constant in m^2^ kg s^-2^ K^-1^, *T* is the temperature in K, and *x* is the extension of the protein chain in m, i.e. the distance between the attachment points of the protein at the tip and the substrate.

### Protein structure prediction

Protein homology modeling of structures of *Mh*Lap RII repeats 2–5 and 21–24 was performed with the freely accessible program Phyre2 (http://www.sbg.bio.ic.ac.uk/~phyre2), in normal mode. [28]

## Supporting information

S1 Supporting Information Amino acid sequences of protein constructs, details of force spectroscopy experiments, details of Phyre2 modelling of *Mh*Lap RII, hydrogen bonds in terminal β-strands of *Mp*AFP RII and other mechanically stable proteins, Matlab script to select H-bonds between terminal strands, differential scanning calorimetry of *Mp*AFP RII at different Ca^2+^ concentrations, GFP unfolding statistics, *Mp*AFP RII_8_-GFP absorbance and fluorescence spectra. and dynamic light scattering of *Mp*AFP RII_8_-GFP and *Mh*Lap RII_8_. (DOCX)
