# Supplementary material for: Unusually high mechanical stability of bacterial adhesin extender domains having calcium clamps
Source: PLoS One. 2017 Apr 4;12(4):e0174682. doi: 10.1371/journal.pone.0174682 (PMC5380327; doi:10.1371/journal.pone.0174682)
Supplement: S1 Supporting Information — (DOCX) [file pone.0174682.s001.docx]

­­Supporting Information

­­­­­Unusually high mechanical stability of bacterial adhesin extender domains having calcium clamps

*Anneloes S. Oude Vrielink, Tyler D. R. Vance, Arthur M. de Jong, Peter L. Davies, Ilja K. Voets*

Contents

[Section A. Protein Constructs 3](#_Toc477450008)

[Amino acid sequence of *Mp*AFP RII_8_-GFP. 3](#_Toc477450009)

[Amino acid sequence of I27^RS^_8_ 4](#_Toc477450010)

[Amino acid sequence of *Mh*Lap RII_8_ 5](#_Toc477450011)

[Section B. Force spectroscopy experiments. 6](#_Toc477450012)

[Unfolding force histograms of *Mp*AFP RII, *Mh*Lap RII and I27. 9](#_Toc477450013)

[Contour length increase and persistence length histograms of *Mp*AFP RII, *Mh*Lap RII and I27 10](#_Toc477450014)

[Contour length increase. 11](#_Toc477450015)

[Pulling speed dependence on loading rate. 12](#_Toc477450016)

[Unfolding histograms of *Mp*AFP RII at different Ca^2+^ concentrations. 13](#_Toc477450017)

[Unfolding force histogram of I27 in buffer with calcium. 14](#_Toc477450018)

[Section C. Structure and topology of *Mh*Lap RII 15](#_Toc477450019)

[Section D. Hydrogen bonds in terminal β-strands of *Mp*AFP RII and other mechanically stable proteins 17](#_Toc477450020)

[Section E. Matlab script to select H-bonds between terminal strands. 21](#_Toc477450021)

[Section F. Reduced thermal stability of *Mp*AFP RII tetra-tandemer in absence of calcium. 22](#_Toc477450022)

[Section G. GFP unfolding statistics. 23](#_Toc477450023)

[Section H. *Mp*AFP RII_8_-GFP absorbance and fluorescence spectra 24](#_Toc477450024)

[Section I. Dynamic light scattering on *Mp*AFP RII_8_-GFP and *Mh*Lap RII_8_ 25](#_Toc477450025)

# Section A. Protein Constructs

Amino acid sequence of *Mp*AFP RII_8_-GFP. *Mp*AFP RII monomers are indicated in cyan and grey and GFP is indicated in green. Compared to wild-type GFP (PDB code 1GFL), this GFP variant bears mutations A1V, F64L, S65T, R80Q and H231L. Un-highlighted sequenced indicates non-native additions, such as His-tags, added codons from restriction enzyme sites, and the C-terminal cysteines for surface attachment. Octa-tandemer sequence was constructed by ligating together two tetra-tandemer genes, previously synthesized and codon optimized for expression in *E. coli* by GeneArt. Both tetra-tandemers were identical, save for their flanking cut sites. The N-terminal tetra-tandemer was flanked immediately up and downstream by *Nde1* and *HindIII* sites, respectively. The C-terminal tetra-tandemer was likewise flanked by *HindIII* and *Xho1* sites. Following sequential ligation into the pET28a expression vector, the internal GFP was added via the internal *HindIII* site between tetra-tandemers. An internal *BseR1* cut site within the first third of the GFP DNA sequence was exploited to determine correct orientation through a double digest with *Xho1* and *BseR1*. Clones containing the correct band length were sent for sequencing (Robarts Research Institute, Western ON).

MASSHHHHHHSSGLVPRGSHMTEATAGTVTVNAITSDDVINASEAAGTVAVSGTATGGDIAEGDTVTLEINGETYTTTVDANGEWSVDVAGSDLAADTAFDAVVTSSDAAGNTVDTTGSSTHTVDTEATAGTVTVNAITSDDVINASEAAGTVAVSGTATGGDIAEGDTVTLEINGETYTTTVDANGEWSVDVAGSDLAADTAFDAVVTSSDAAGNTVDTTGSSTHTVDTEATAGTVTVNAITSDDVINASEAAGTVAVSGTATGGDIAEGDTVTLEINGETYTTTVDANGEWSVDVAGSDLAADTAFDAVVTSSDAAGNTVDTTGSSTHTVDTEATAGTVTVNAITSDDVINASEAAGTVAVSGTATGGDIAEGDTVTLEINGETYTTTVDANGEWSVDVAGSDLAADTAFDAVVTSSDAAGNTVDTTGSSTHTVDKLMVSKGEELFTGVVPILVELDGDVNGHKFSVSGEGEGDATYGKLTLKFICTTGKLPVPWPTLVTTLTYGVQCFSRYPDHMKQHDFFKSAMPEGYVQERTIFFKDDGNYKTRAEVKFEGDTLVNRIELKGIDFKEDGNILGHKLEYNYNSHNVYIMADKQKNGIKVNFKIRHNIEDGSVQLADHYQQNTPIGDGPVLLPDNHYLSTQSALSKDPNEKRDHMVLLEFVTAAGITLGMDELYKKLTEATAGTVTVNAITSDDVINASEAAGTVAVSGTATGGDIAEGDTVTLEINGETYTTTVDANGEWSVDVAGSDLAADTAFDAVVTSSDAAGNTVDTTGSSTHTVDTEATAGTVTVNAITSDDVINASEAAGTVAVSGTATGGDIAEGDTVTLEINGETYTTTVDANGEWSVDVAGSDLAADTAFDAVVTSSDAAGNTVDTTGSSTHTVDTEATAGTVTVNAITSDDVINASEAAGTVAVSGTATGGDIAEGDTVTLEINGETYTTTVDANGEWSVDVAGSDLAADTAFDAVVTSSDAAGNTVDTTGSSTHTVDTEATAGTVTVNAITSDDVINASEAAGTVAVSGTATGGDIAEGDTVTLEINGETYTTTVDANGEWSVDVAGSDLAADTAFDAVVTSSDAAGNTVDTTGSSTHTVDCC

Amino acid sequence of I27^RS^_8_**.** I27 monomers are indicated in cyan. The gene I27^RS^_8_ was constructed according to work of Carrion-Vazquez *et al*, [[1](#_ENREF_1)] synthesized and cloned into pET15b by GenScript.

MGSSHHHHHHSSGLVPRGSHMLIEVEKPLYGVEVFVGETAHFEIELSEPDVHGQWKLKGQPLTASPDCEIIEDGKKHILILHNCQLGMTGEVSFQAANAKSAANLKVKELRSLIEVEKPLYGVEVFVGETAHFEIELSEPDVHGQWKLKGQPLTASPDCEIIEDGKKHILILHNCQLGMTGEVSFQAANAKSAANLKVKELRSLIEVEKPLYGVEVFVGETAHFEIELSEPDVHGQWKLKGQPLTASPDCEIIEDGKKHILILHNCQLGMTGEVSFQAANAKSAANLKVKELRSLIEVEKPLYGVEVFVGETAHFEIELSEPDVHGQWKLKGQPLTASPDCEIIEDGKKHILILHNCQLGMTGEVSFQAANAKSAANLKVKELRSLIEVEKPLYGVEVFVGETAHFEIELSEPDVHGQWKLKGQPLTASPDCEIIEDGKKHILILHNCQLGMTGEVSFQAANAKSAANLKVKELRSLIEVEKPLYGVEVFVGETAHFEIELSEPDVHGQWKLKGQPLTASPDCEIIEDGKKHILILHNCQLGMTGEVSFQAANAKSAANLKVKELRSLIEVEKPLYGVEVFVGETAHFEIELSEPDVHGQWKLKGQPLTASPDCEIIEDGKKHILILHNCQLGMTGEVSFQAANAKSAANLKVKELRSLIEVEKPLYGVEVFVGETAHFEIELSEPDVHGQWKLKGQPLTASPDCEIIEDGKKHILILHNCQLGMTGEVSFQAANAKSAANLKVKELRSCC

Amino acid sequence of *Mh*Lap RII_8_**.** *Mh*Lap RII monomers are indicated in cyan and grey. As with *Mp*AFP, the octa-tandemer was constructed using two tetra-tandemer building blocks previously sequenced by GeneArt. In this case, the N-terminal tetra-tandemer contained sequence corresponding to repeats 2-5 from the native protein, while the C-terminal tetra-tandemer contained repeats 21-24. The same cut sites were used to ligate the genes together in pET28a as with the *Mp*AFP construct.

MASSHHHHHHSSGLVPRGSHMSFDATAGALTVSLDTVDNTAQTANLSGTTTDVAPNEQVAITITDSAGNIVNAIATVGADGSYSLTGVDISSLVDGSLTVEASAQDRNGNALTDSANGALDATAGDLTVSVGTIDNTAQTVNLSGTTTDVAPNGQVAITMTDSAGNIVNATATVGADGSYSLTGVDISSLVDGDLTVEASAQDRNGNAVSDSANGTFDATAGDLTVSVDTVDSTAQTANLSGTTTDVALNSQVDLTVTDSAGNVVTATTTVGADGSYSLTGVDISSLVDGNLTVEATAQDRNGNAVSDSAAGSLDATTGALTVSLDTVDNAAQTVDLSGTTADVAPNSQVNVTITDSTGNVVNAITTVGADGSYSLTGVDISSLVDGDLTVEASAQGRNGNALTDSANGALKLDATAGDLTVSIATIDNGNQTINLSGTTTDVAPNSQVEVTITDSAGNVVNATATVGANGSYFLTGVDISRLVDGSLTVEALAQDRNGNAVSDSANGTFDATAGDLTVSVDTVDNTAQTVNLSGTTTDVAPNGQIAITITDSAGNAVNATTTVDADGAYTLTGVDISRLVDGNLTIEATAQDRNGNAVSDSAAGSLDATTGDLTVAIANVDNGNQTADLSGSTTDVAPNSQVNVTITDSAGSTVTATAIVGADGSYTLSGVDISSLVDGDLTAEASAQDRNGNHVSDSVTGSFDATAGDLAVTISNVDNGAQTIDLSGTTTDVAPNSEVEVTITDSAGNVVNTTATVDADGSYTLTGVDIASLVDGNLTVEATAQDRNGNAVSDSANGTFDATCC

Sequence identity (%) between repeats in construct *Mh*Lap RII_8._ On average there is 78.2% sequence identity between two repeats in the construct and 77.6% identity between two subsequent repeats. In the full *Mh*Lap Region II there is on average 75.8% sequence identity between subsequent repeats.

**Table A.** *Sequence identity (%) between repeats in MhLap RII_8_.*

| % | 2 | 3 | 4 | 5 | 21 | 22 | 23 | 24 |
| --- | --- | --- | --- | --- | --- | --- | --- | --- |
| 2 |  | 86.6 | 81.4 | 86.6 | 79.4 | 80.4 | 70.1 | 74.2 |
| 3 |  |  | 82.5 | 78.4 | 84.5 | 83.5 | 83.5 | 78.4 |
| 4 |  |  |  | 77.3 | 78.4 | 84.5 | 72.2 | 76.3 |
| 5 |  |  |  |  | 75.3 | 74.2 | 71.1 | 73.2 |
| 21 |  |  |  |  |  | 77.3 | 76.3 | 83.5 |
| 22 |  |  |  |  |  |  | 69.1 | 76.3 |
| 23 |  |  |  |  |  |  |  | 75.3 |
| 24 |  |  |  |  |  |  |  |  |

# Section B. Force spectroscopy experiments.

Force curve analysis was performed with PUNIAS software. Unfolding forces were determined as the height of the protein unfolding peak minus the height of the baseline. The unfolding length $\Delta L$ was determined from the difference in position between subsequent peaks (Figure 2). The contour length increase $\Delta L_{c}$, and persistence length $L_{p}$ were determined from fits of the worm-like chain (WLC) model to protein unfolding peaks, $F\left( x \right)=\left( \frac{k_{B}T}{L_{p}} \right)\left[ \frac{1}{4\left( 1-\frac{x}{L_{c}} \right)^{2}}-\frac{1}{4}+\frac{x}{L_{c}} \right]$ where $F\left( x \right)$ is the force (N), $L_{c}$ is the contour length of the stretched protein (m), $L_{p}$ is the persistence length (m), $k_{B}$ is the Boltzmann constant (m^2^ kg s^-2^ K^-1^), $T$ is the temperature (K), and $x$ is the extension of the protein chain, i.e. the distance between the attachment points of the protein (m).

**Table B.** *MpAFP RII_8_-GFP – pulling speed dependence*

| Speed  (nm/s) | N | *k* (N/m) | Hit rate^†^ (%) | Average  (±S.D.) Force (pN) | Average (±S.D.) ^‡^ Force (pN) | Average ∆L (±S.D.) (nm) | Average ∆L_C_ (±S.D.) (nm) | Average ∆L_C_ (±S.D.) (nm) | Average L_p_ (±S.D.)  (nm) |
| --- | --- | --- | --- | --- | --- | --- | --- | --- | --- |
| 50 | 56 | 0,14 | 1,1 | 306 (30) | 289 (37) | 29,7 (1,9) | 32,2 (2,3) | 32,4 (2,9) | 0,33 (0,1) |
| 50 | 43 | 0,14 | 4,6 | 265 (42) |  | 29,3 (2,5) | 32,2 (2,4) |  | 0,47 (0,3) |
| 50^*^ | 51 | 0,14 | < 1,0 | 297 (37) |  | 30,3 (1,7) | 32,6 (3,8) |  | 0,26 (0,2) |
| 200 | 70 | 0,14 | 4,4 | 347 (59) | 341 (55) | 29,5 (2,1) | 31,8 (4,8) | 33,2 (3,7) | 0,31(0,3) |
| 200 | 40 | 0,21 | 1,5 | 344 (52) |  | 31,0 (1,6) | 34,7 (3,6) |  | 0,17 (0,1) |
| 200^*^ | 60 | 0,21 | < 1,0 | 332 (55) |  | 30,2 (1,4) | 33,0 (2,4) |  | 0,29 (0,2) |
| 1000 | 352 | 0,12 | 8,1 | 326 (32) | 348 (37) | 33,0 (7,0) | 33,5 (2,7) | 33,2 (2,8) | 0,32 (0,1) |
| 1000 | 109 | 0,12 | 12,9 | 318 (33) |  | 32,0 (3,0) | 32,8 (3,1) |  | 0,30 (0,2) |
| 1000 | 57 | 0,15 | 1,7 | 399 (45) |  | 31,5 (3,8) | 33,2 (2,6) |  | 0,23 (0,1) |
| 4880 | 43 | 0,13 | 2,7 | 408 (38) | 377 (39) | 32,1 (2,4) | 33,8 (4,6) | 32,7 (4,0) | 0,20 (0,1) |
| 4880 | 99 | 0,14 | 1,8 | 362 (37) |  | 31,1 (1,9) | 32,9 (2,7) |  | 0,34 (0,1) |
| 4880 | 131 | 0,14 | 3,0 | 360 (42) |  | 30,3 (5,5) | 31,5 (4,3) |  | 0,31 (0,1) |

**Table C.** *MpAFP RII_8_-GFP – Ca^2+^ dependence (1 µm/s pulling speed)*

| Conc. free Ca^2+^ | N | *k* (N/m) | Hit rate^†^ (%) | Average  (±S.D.) Force (pN) | Average (±S.D.) ^‡^ Force (pN) | Average ∆L (±S.D.) (nm) | Average ∆L_C_ (±S.D.) (nm) | Average ∆L_C_ (±S.D.) (nm) | Average L_p_ (±S.D.)  (nm) |
| --- | --- | --- | --- | --- | --- | --- | --- | --- | --- |
| 30 µM | 73 | 0,13 | 1,3 | 218 (53) | 245 (54) | 30,7 (3,7) | 32,6 (3,7) | 32,8 (4,7) | 0,42 (0,2) |
| 30 µM | 57 | 0,15 | 1,7 | 240 (68) |  | 30,9 (6,8) | 32,2 (5,4) |  | 0,45 (0,3) |
| 30 µM | 50 | 0,16 | 1,2 | 276 (50) |  | 30,7 (4,8) | 33,6 (4,9) |  | 0,54 (0,3) |
| 10 mM | 62 | 0,16 | 1,0 | 379 (44) | 367 (54) | 30,1 (1,8) | 33,1 (1,7) | 32,4 (1,8) | 0,30 (0,1) |
| 10 mM | 43 | 0,15 | 1,5 | 354 (62) |  | 29,8 (2,7) | 31,8 (1,9) |  | 0,30 (0,1) |

**Table D.** *MhLap RII_8_*

| Speed  (nm/s) | N | *k* (N/m) | Hit rate^†^ (%) | Average  (±S.D.) Force (pN) | Average (±S.D.) ^‡^ Force (pN) | Average ∆L (±S.D.) (nm) | Average ∆L_C_ (±S.D.) (nm) | Average ∆L_C_ (±S.D.) (nm) | Average L_p_ (±S.D.)  (nm) |
| --- | --- | --- | --- | --- | --- | --- | --- | --- | --- |
| 1000 | 94 | 0,24 | 1,2 | 365 (57) | 306 (51) | 29,8 (1,9) | 32,6 (3,8) | 33,6 (4,6) | 0,13 (0,0) |
| 1000 | 306 | 0,20 | 3,9 | 272 (42) |  | 30,5 (2,4) | 33,7 (5,3) |  | 0,21 (0,0) |
| 1000 | 76 | 0,22 | 3,8 | 269 (45) |  | 30,1 (1,9) | 34,5 (6,7) |  | 0,24 (0,1) |
| 1000 | 254 | 0,20 | 4,1 | 310 (60) |  | 30,0 (2,1) | 33,6 (2,7) |  | 0,19 (0,0) |
| 1000 | 276 | 0,23 | 4,2 | 316 (47) |  | 29,8 (2,0) | 33,6 (3,1) |  | 0,20 (0,0) |

**Table E.** *I27^RS^_8_ (experiments highlighted in green were performed in 50 mM Tris-HCl, 200 mM NaCl, 10 mM CaCl_2_, pH 9, non-highlighted experiments were performed in PBS buffer)*

| Speed  (nm/s) | N | *k* (N/m) | Hit rate^†^ (%) | Average  (±S.D.) Force (pN) | Average (±S.D.) ^‡^ Force (pN) | Average ∆L (±S.D.) (nm) | Average ∆L_C_ (±S.D.) (nm) | Average ∆L_C_ (±S.D.) (nm) | Average L_p_ (±S.D.)  (nm) |
| --- | --- | --- | --- | --- | --- | --- | --- | --- | --- |
| 1000 | 66 | 0,19 | 1,3 | 204 (26) | 218 (29) | 25,2 (1,8) | 27,3 (4,8) | 27,3 (5,3) | 0,3 (0,1) |
| 1000 | 60 | 0,19 | 4,0 | 241 (27) |  | 25,4 (2,4) | 26,9 (7,2) |  | 0,2 (0,1) |
| 1000 | 159 | 0,22 | 2,8 | 200 (30) |  | 25,9 (4,5) | 27,3 (5,2) |  | 0,3 (0,1) |
| 1000 | 63 | 0,19 | 2,5 | 225 (32) |  | 25,4 (2,1) | 27,6 (2,9) |  | 0,3 (0,1) |
| 1000 | 22 | 0,19 | 0,7 | 223 (33) | 231 (38) | 25,1 (2,2) | 25,5 (4,4) | 26,1 (6,4) | 0,3 (0,1) |
| 1000 | 52 | 0,21 | 2,5 | 219 (36) |  | 24,7 (2,6) | 27,1 (2,7) |  | 0,4 (0,2) |
| 1000 | 58 | 0,19 | 4,0 | 242 (44) |  | 25,0 (2,7) | 27,1 (5,3) |  | 0,2 (0,1) |
| 1000 | 48 | 0,20 | 2,8 | 222 (38) |  | 24,3 (2,3) | 23,9 (7,3) |  | 0,2 (0,1) |
| 1000 | 41 | 0,21 | 4,0 | 244 (25) |  | 25,5 (2,1) | 26,9 (9,9) |  | 0,2 (0,1) |

^*^ Result is a combination of three experiments, performed on different days with different cantilevers (average spring constant is given in the table).

^†^  Percentage of total number of force-extension profiles that remain after filtering the data for curves where minimum of three protein unfolding events were observed.

^‡^ Standard deviation (S.D.) of $n$ combined data sets is calculated according to $\sigma_{n}=\sqrt{\frac{\sigma_{1}^{2}+\sigma_{2}^{2}+\sigma_{3}^{2}+{\ldots+\sigma}_{n}^{2}}{n}}$

Unfolding force histograms of *Mp*AFP RII, *Mh*Lap RII and I27. Lines of different colors represent distributions of replicate pulling experiments. For each replicate experiment a sample solution was prepared freshly and a different cantilever was used. For the rest, all experiments were measured under identical conditions unless noted otherwise. Pulling experiments of *Mp*AFP RII and *Mh*Lap RII were performed in 50 mM Tris-HCl (pH 9), 200 mM NaCl, 10 mM CaCl_2_, 3 mM TCEP. Pulling experiments with I27 were performed in PBS buffer. Unfolding force and unfolding length distributions of pulling experiments of I27 in 50 mM Tris-HCl (pH 9), 200 mM NaCl and 10 mM CaCl_2_ are shown in Figure E. Furthermore, for each pulling experiment at least 50 protein unfolding peaks were observed.

**
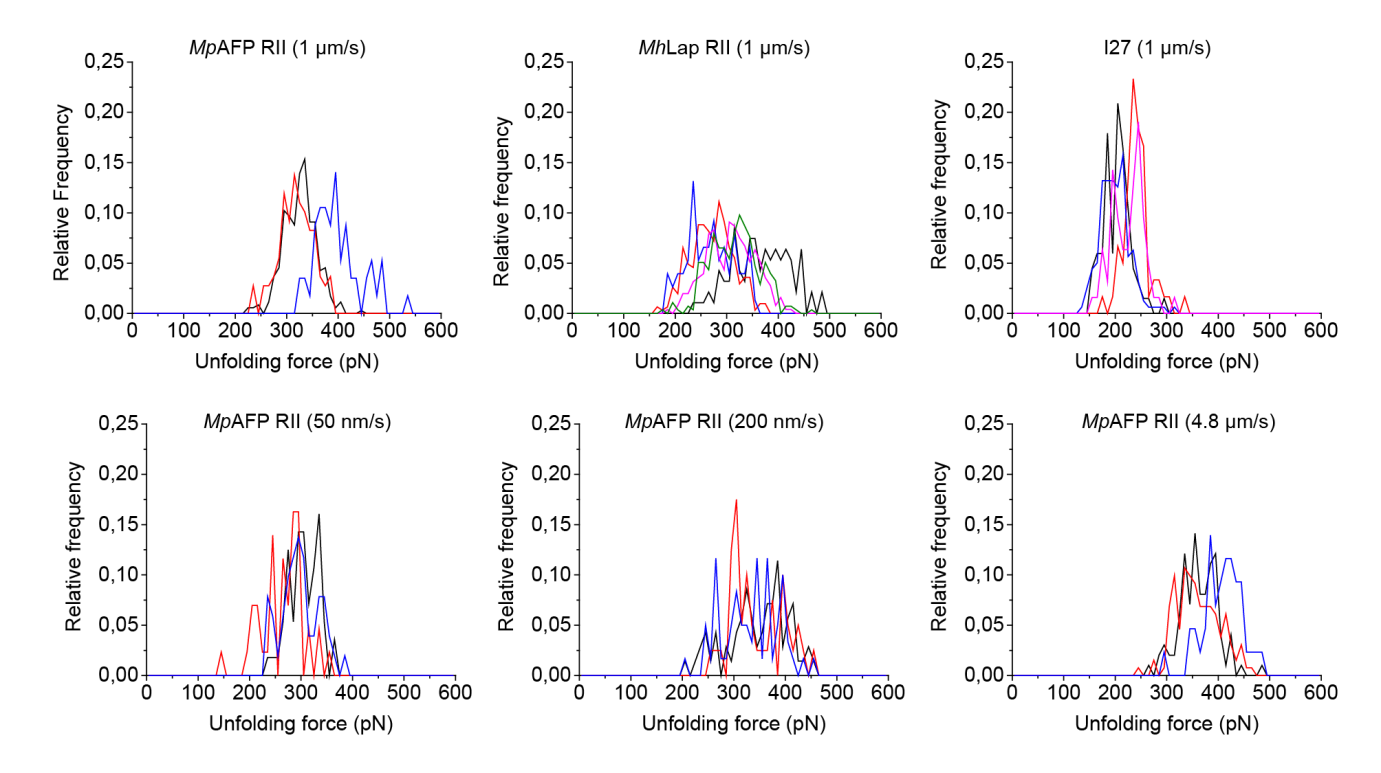
 Figure A.** *Normalized distribution of measured unfolding forces for MpAFP RII, MhLap RII and I27 at 1 µm/s pulling speed, and for MpAFP at 50 nm/s, 200 nm/s and 4.8 µm/s pulling speed. Bin size is 10 pN. Details of experiments are shown in Table B-E.*

Contour length increase and persistence length histograms of *Mp*AFP RII, *Mh*Lap RII and I27**.** Lines of different colors represent distributions of replicate pulling experiments, for each pulling experiment at least 50 protein unfolding peaks were observed.

**
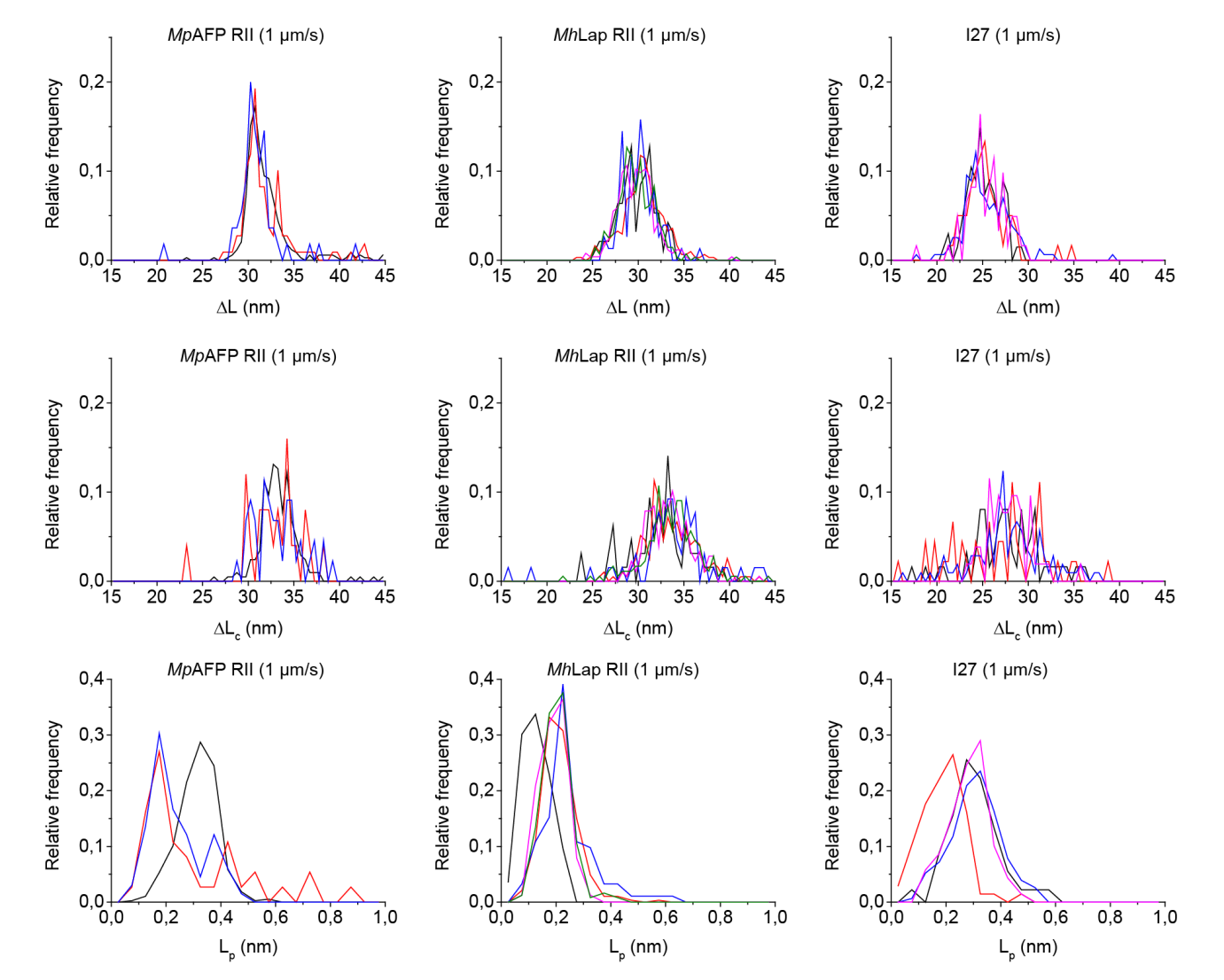
**

**Figure B.** *Normalized distribution of measured unfolding lengths ∆L, contour length increases ∆L_c_, and persistence lengths L_p_; for MpAFP RII, MhLap RII and I27 at 1 µm/s pulling speed. Bin size of ∆L and ∆L_c_ is 0.5 nm, and bin size of L_p_ is 0.05 nm. Details of experiments are shown in Table B-E.*

Contour length increase. The contour length increase per amino acid was determined for each protein based on experiments with 1 µm/s pulling speed.

**Table F.** *Contour length increases.*

| **Protein** | **∆*L*_c_ (nm)** | ***d*_N;C_ (nm)** | ***L*_c,aa_ (nm/aa)** |
| --- | --- | --- | --- |
| *Mp*AFP RII | 33,2 | 4,81 | (33,2 + 4,81) / 104 = 0,37 |
| *Mh*Lap RII | 33,6 | 3,38 | (33,6 + 3,38) / 97 = 0,38 |
| I27 | 27,3 | 4,32 | (27,3 + 4,32) / 91 = 0,35 |

**∆***L*_c_ **=** contour length increase, *d*_N;C_ ***=*** N- to C- terminal distance of folded monomer,

*L*_c,aa_ = contour length increase per amino acid.

Pulling speed dependence on loading rate. In Figure S4 the dependence of the unfolding force on the loading rate is presented. The data were fitted by a linear function $F \left( k_{c}v \right)=\frac{k_{B}T}{x_{u}}\ln\left( \frac{k_{c}vx_{u}}{k_{B}Tk_{u}^{0}} \right),$ [[2](#_ENREF_2)] from which the unfolding potential width $x_{u}$ ~ 0.2 nm and unfolding rate constant at zero force $k_{u}^{0}$ ~ 0.003 s^-1^ could be estimated from the slope and intercept, respectively.

**
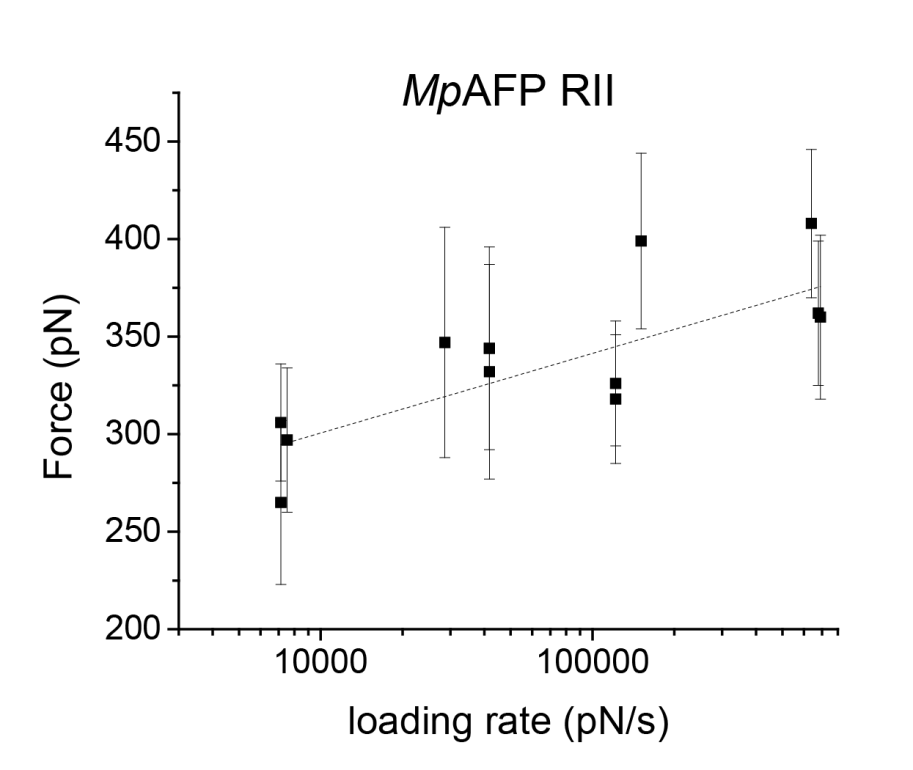
**

**Figure C.** *MpAFP RII unfolding forces (pN) as function of the loading rate (pN/s). At each pulling velocity (50, 200, 1000 and 4880 nm/s) three experiments with three different cantilevers were performed. Each experiment contains at least 50 protein unfolding peaks.*

# **Unfolding histograms of *Mp*AFP RII at different Ca^2+^ concentrations.**

From the unfolding force histograms of *Mp*AFP RII at 30 µM and 10 mM Ca^2+^ it can be observed that unfolding forces shift to lower values at 30 µM Ca^2+^ compared to 10 mM Ca^2+^. Thus, Ca^2+^ is important for the mechanical stability of *Mp*AFP RII. The histograms in Figure S5B and S4C were obtained by first dialyzing *Mp*AFP RII_8_-GFP to a buffer with 0.1 mM EDTA. Subsequently, Ca^2+^ was added to a free concentration of either 30 µM or 10 mM. Importantly, the depletion of Ca^2+^ in the dialysis step did not irreversibly destabilize the protein, as the *Mp*AFP RII unfolding forces histogram obtained after addition of 10 mM Ca^2+^ (Figure S5C) were comparable to unfolding force histograms obtained in 10 mM Ca^2+^ with *Mp*AFP RII that had not been dialyzed to Ca^2+^-free buffer (Figure S5A). **
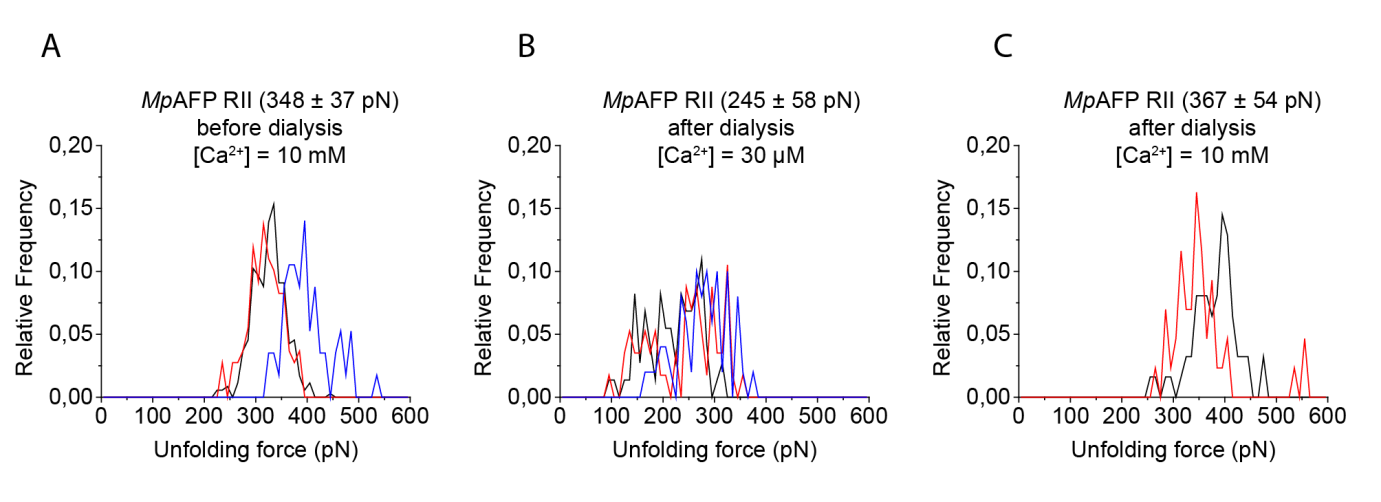
Figure D.** *Unfolding force histograms of non-dialyzed MpAFP RII at 10 mM Ca^2+^ (A, three experiments; N = 352, 109 and 57), MpAFP RII at 30 µM Ca^2+^ (B, three experiments; N = 73, 57 and 50) and MpAFP RII at 10 mM Ca^2+^ after dialysis (C, two experiments, N = 62 and 43). Lines of different colors represent the unfolding force histograms of replicate experiments. At lower Ca^2+^ concentration, MpAFP RII unfolding forces shift to lower values. Addition of 10 mM Ca^2+^ to MpAFP in Ca^2+^-free buffer fully recovers the stable Ca^2+^-bound fold. ­­*

# **Unfolding force histogram of I27 in buffer with calcium.**

To verify that the enhancement of the mechanical stability in *Mp*AFP RII is a specific effect due to calcium binding the effect of calcium concentration on the stability of I27 was determined. Force measurements of I27_RS_^8^ were performed in 50 mM Tris-HCl (pH 9), 200 mM NaCl, 10 mM CaCl_2_, with 1 µm/s pulling speed. On average, an unfolding force *F*_u_ = 231 ± 38 pN (N = 221) was obtained. This is comparable to the unfolding force obtained in PBS buffer which does not contain calcium; *F*_u_ = 218 ± 29 pN (N = 348). We conclude that calcium does not significantly influence the mechanical stability of I27.


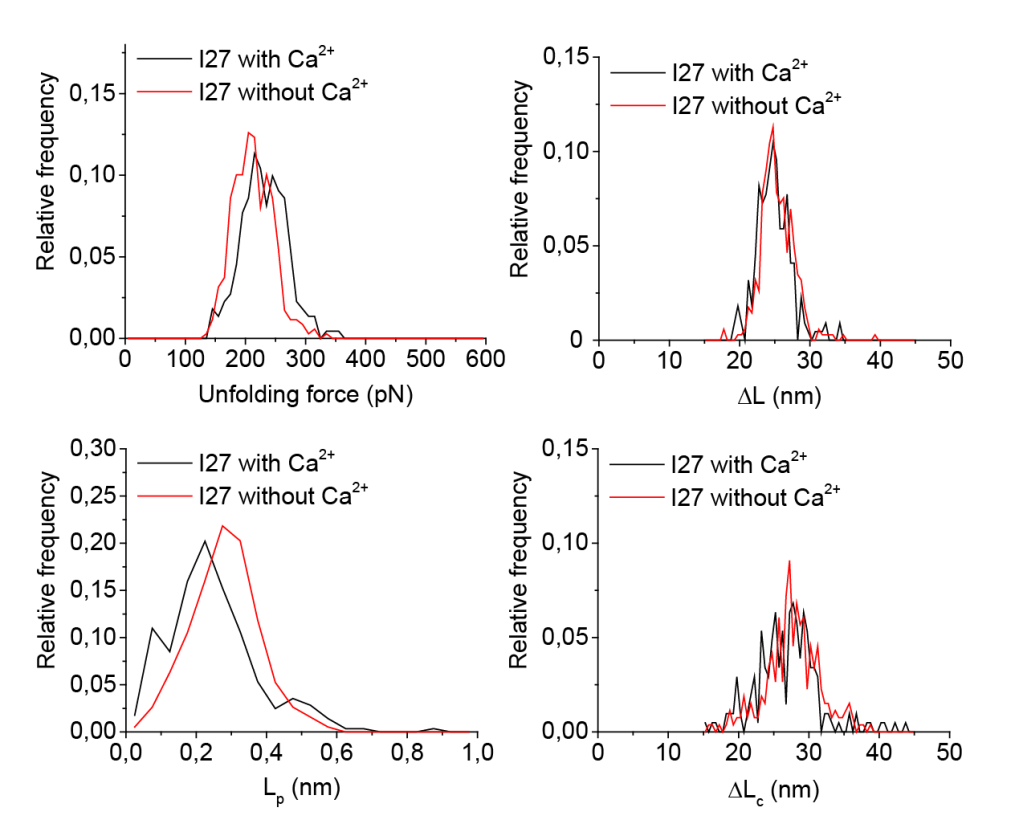


**Figure E.** *Unfolding force, unfolding length, persistence length and contour length increase histograms of I27^RS^_8_ in buffer with calcium (50 mM Tris-HCl, 200 mM NaCl, 10 mM CaCl_2_, pH 9) and buffer without calcium (PBS buffer; 137 mM NaCl, 2.7 mM KCl, 10 mM Na_2_HPO_4_, 1.8 mM KH_2_PO_4_, pH 7.4). The unfolding force histograms appear very similar indicating that calcium does not significantly increase the mechanical stability of I27.*

# Section C. Structure and topology of *Mh*Lap RII

The structure of repeats 2-5 and 21-24 of *Mh*Lap RII have been predicted by Phyre2 homology modeling (<http://www.sbg.bio.ic.ac.uk/~phyre2>) (Figure S7). [[3](#_ENREF_3)] Interestingly, *Mp*AFP RII *Mp*AFP RII monomer (4kdw) and *Mp*AFP RII tetra-tandemer (PDB 4p99) were identified as template proteins with the highest confidence (Table S2). The confidence represents the probability that the match between the sequence and template is true homology. Homologous proteins are extremely likely to adopt similar structures and often similar functions. Generally, for matches with confidence > 90% the protein adopts the overall fold that is modelled and the core of the protein has 2 – 4 Å root mean square deviation from the native, true structure. [[4](#_ENREF_4)]

####
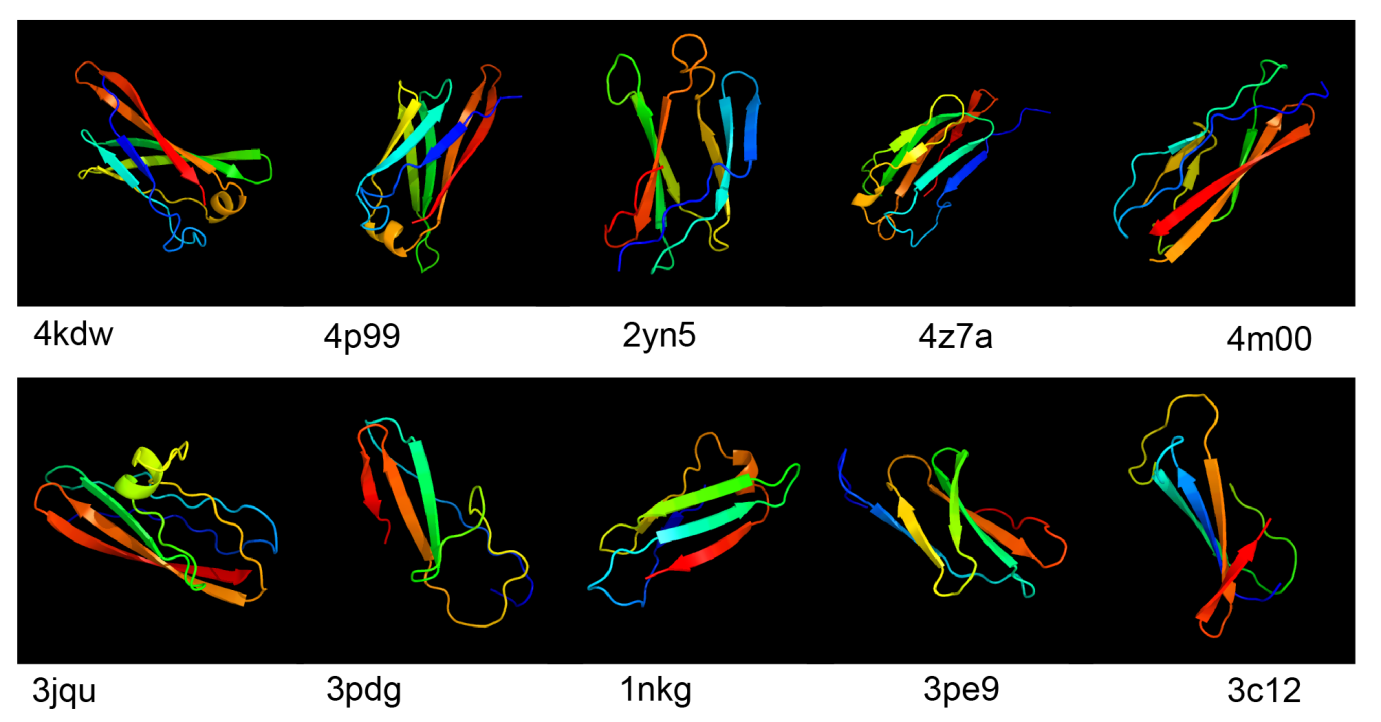


**Figure F.** *Phyre2 homology models of MhLap RII repeat 2 based on ten templates with highest sequence confidence.*

**Table G.** *Phyre2 templates with the highest average confidence for modeling MpAFP RII repeats 2 – 5 and 21 – 24. MpAFP RII monomer and tetra-tandemer crystal structures had the highest confidence over all repeats indicating that the proteins are homologous. Furthermore, other bacterial adhesins SiiE and SraP were selected, as well as other Ca^2+^ binding proteins.*

| **PDB** | **Protein** | **Sequence**  **identity (%)** | **confidence (%)** |
| --- | --- | --- | --- |
| 4kdw | *Mp*AFP RII monomer (Ca^2+^ binding) | 33.1 ± 3.1 | 99.6 ± 0.1 |
| 4p99 | *Mp*AFP RII tetra-tandemer (Ca^2+^ binding) | 31.3 ± 5.1 | 98.8 ± 0.2 |
| 2yn5 | bacterial adhesin SiiE (Ca^2+^ binding) | 23.3 ± 2.3 | 98.0 ± 0.3 |
| 4z7a | IdtMt5 transpeptidase | 18.6 ± 7.8 | 96.9 ± 0.5 |
| 3jqu | ColG collagenase (Ca^2+^ binding) | 13.8 ± 2.1 | 95.1 ± 1.4 |
| 3pdg | cellobiohydrolase A (CbhA) (Ca^2+^ binding) | 17.0 ± 1.9 | 94.9 ± 1.2 |
| 3pe9 | cellobiohydrolase A (CbhA) (Ca^2+^ binding) | 19.1 ± 4.1 | 94.9 ± 1.1 |
| 3c12 | flagellar protein FlgD | 20.3 ± 2.5 | 94.4 ± 1.1 |
| 1nkg | RG-lyase | 14.9 ± 2.9 | 94.3 ± 1.0 |
| 4m00 | bacterial adhesion protein SraP (Ca^2+^ binding) | 17.6 ± 1.3 | 94.3 ± 3.6 |

####

# Section D. Hydrogen bonds in terminal β-strands of *Mp*AFP RII and other mechanically stable proteins

Overview of hydrogen- and ionic bonds that stabilize the fold of *Mp*AFP RII. Hydrogen bonds between terminal strands were predicted from the crystal structure of *Mp*AFP RII tetra-tandemer (PDB file 4P99). The number of hydrogen bonds in *Mp*AFP RII repeats is not large compared to the number of H-bonds observed for other mechanically stable proteins, which can be observed in Table S10.

**
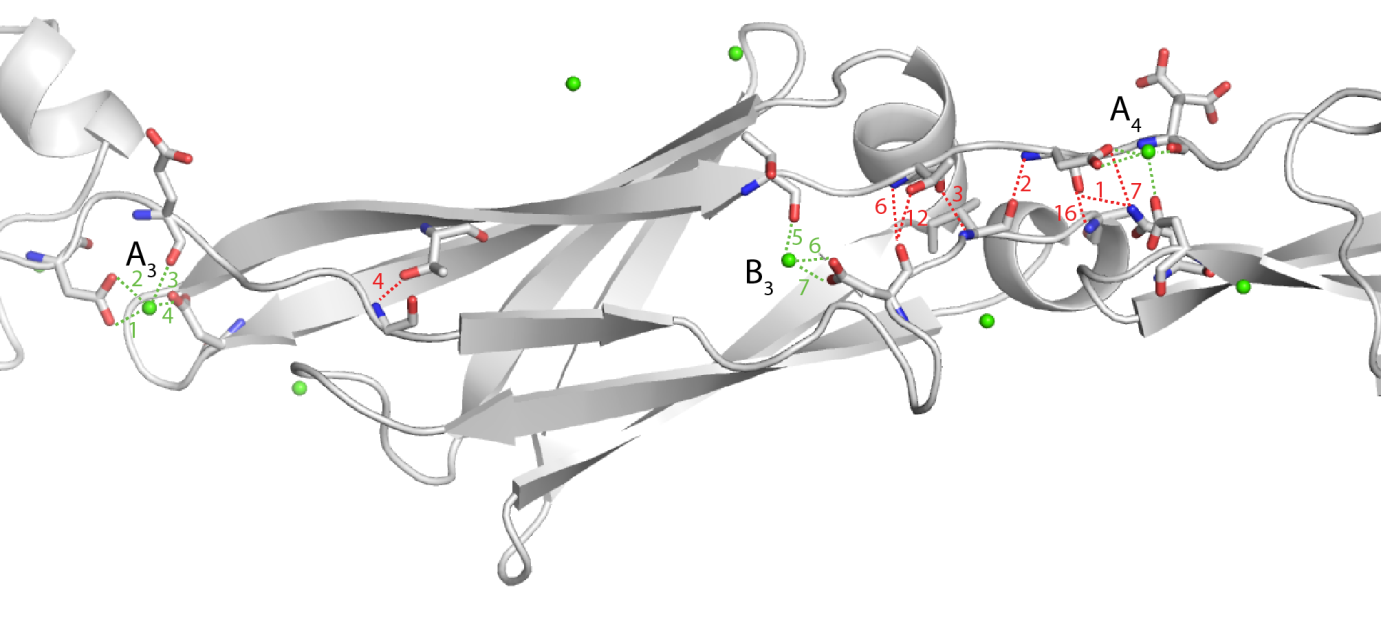
**

**Figure G.** *Frequently occuring hydrogen bonds of MpAFP RII are indicated in repeat 3 of chain A of MpAFP RII tetra-tandemer by red dashed lines (4P99). Numbering of H-bond­­­­s corresponds to numbering in table S8. The hydrogen bonds 1 and 7 are in between subsequent repeats. Ca^2+^ ions are indicated as green spheres, and ionic interactions between residues and Ca^2+^ ions are indicated with green dashed lines. The numbering of the ionic bonds corresponds to the numbering in table S9. The numbering of the calcium ions refers to the number of the repeat in the tetra-tandemer (e.g. A3 corresponds to calcium ion A in the third repeat).*

**Table H.** *Hydrogen bonds in MpAFP RII tetra-tandemer (PDB code 4P99) between terminal strands. The hydrogen bonds D – H ··· A are ordered in increasing distances between the donor D and acceptor atom A. No constraints were applied to the bond angles. Several hydrogen bonds form between subsequent repeats (1, 7, 8, 10 and 18). The abundance of a particular hydrogen bond is calculated from the number of times the hydrogen bond of distance less than 4 Å occurred in four tetra-tandemers in the crystal unit cell, divided by 16 (for intra-repeat hydrogen bonds) or 12 (for inter-repeat hydrogen bonds). Hydrogen bonds with distances shorter than 3.2 Å are indicated in green, other hydrogen bonds with high probability are indicated in brown. Atoms of amino acids are indicated in CYANA nomenclature.*

| **Number** | **Residues** | **Abundance (%)** | **Distance (Å)** |
| --- | --- | --- | --- |
| 1 | Asn91(n+1):ND2 - Asp104(n):O | 100 (N = 12) | 2.8 ± 0.2 |
| 2 | Asp104:N - Ile19:O | 100 (N = 16) | 2.9 ± 0.1 |
| 3 | Ile19:N - Thr102:O | 100 (N = 16) | 2.9 ± 0.1 |
| 4 | Gly6:N - Thr95:OG1 | 100 (N=16) | 3.0 ± 0.1 |
| 5 | Ala21:N - Thr105:OG1 | 6 (N=1) | 3.2 |
| 6 | Thr102:N - Asp17:O | 100 (N=16) | 3.3 ± 0.1 |
| 7 | Asn91(n+1):ND2 - Asp104(n):OD1 | 100 (N=12) | 3.3 ± 0.4 |
| 8 | Thr1(n+1):OG1 - Ile19(n):O | 6 (N=1) | 3.4 |
| 9 | Asn91:ND2 - Glu2:N:B | 13 (N=2) | 3.5 ± 0.04 |
| 10 | Thr1(n+1):OG1 - Asn20(n):O | 17 (N=2) | 3.6 ± 0.5 |
| 11 | Glu2:N - Asn91:OD1 | 13 (N=2) | 3.6 ± 0.4 |
| 12 | Thr102:OG1 – Asp17:O | 88 (N=14) | 3.7 ± 0.2 |
| 13 | Asn91:ND2 - Thr1:O | 25 (N=4) | 3.7 ± 0.1 |
| 14 | Asn91:ND2 - Glu2:O:B | 13 (N=2) | 3.8 ± 0.2 |
| 15 | Asn91:ND2 - Glu2:O | 44 (N=7) | 3.9 ± 0.1 |
| 16 | Ala21:N - Asp104:O | 63 (N=10) | 3.9 ± 0.1 |
| 17 | Gly97:N - Val8:O | 25 (N=4) | 3.9 ± 0.1 |
| 18 | Ala21(n):N - Thr1(n+1):OG1 | 8 (N=1) | 4.0 |

**Table I.** *Calcium-ion coordinating interactions in MpAFP RII, of calcium ions A and B, based on the crystal structure of the tetra-tandemer (4P99). Atoms of amino acids are indicated in CYANA nomenclature. Distances were determined from the four repeats of tetra-tandemers A and B. The ionic interactions 1 and 2 lie between repeats, and the probability takes this into account.*

| **Number** | **Residues** | **Abundance (%)** | **Distance (Å)** |
| --- | --- | --- | --- |
| 1 | Asp104(n-1):OD2 – Ca (A) | 100 (N=6) | 2.49 ± 0.07 |
| 2 | Asp104(n-1):OD1 – Ca (A) | 100 (N=6) | 2.36 ± 0.04 |
| 3 | Glu2:O – Ca (A) | 100 (N=8) | 2.41 ± 0.12 |
| 4 | Asp87:OD2 – Ca (A) | 100 (N=8) | 2.43 ± 0.08 |
| 5 | Thr100:O – Ca (B) | 100 (N=8) | 2.30 ± 0.04 |
| 6 | Asp17: OD2 – Ca (B) | 100 (N=8) | 2.48 ± 0.07 |
| 7 | Asp17:OD1 – Ca (B) | 100 (N=8) | 2.52 ± 0.07 |

**Table J.** *Overview of hydrogen bonds between terminal strands in proteins with N- and C-termini pointing in different directions. The distance between donor atom D and acceptor atom A in hydrogen bond D – H ··· A was classified in three categories. [*[*5*](#_ENREF_5)*] No constraints were applied to the bond angles. The amino acids of the N- and C-terminal strands are indicated. For the MpAFP RII tetra-tandemer (4P99), the amino acids selected to construct the hydrogen bonds were 1-21 (N-) and 89-108 (C-) for the first repeat, 102-125 (N-) and 193-211 (C-) for the second repeat, 207-229 (N-) and 297-316 (C-) for the third repeat, 311-332 (N-) and 401-416 (C-) for the fourth repeat.*

| **PDB** | **Protein** | **Clamp motif** *^(6)^* | ***F*_u_ (pN) /**  ***v* (nm/s)** | **N-** | **C-** | **Number of H-bonds**  **A / B / C**  **A: 2.2 – 2.5 Å**  **B: 2.5 – 3.2 Å**  **C: 3.2 – 4.0 Å** |
| --- | --- | --- | --- | --- | --- | --- |
| 4KDV | *Mp*AFP RII monomer |  | 348 / 1000 | 3 - 20 | 91 - 104 | 0 / 4 / 1 |
| 4P99 | *Mp*AFP RII tetra-tandemer |  | 348 / 1000 |  |  | 0 / 4 / 5.2 |
| Phyre2 model | *Mh*Lap RII, repeat 3 |  | 306 / 1000 | 101-121 | 184 - 201 | 0 / 5 / 5 |
| 1AOH | c7A | SD1 | 510 / 600[[6](#_ENREF_6)] | 5 - 17 | 133 - 147 | 0 / 9 / 4 |
| 1G1K | c1C | SD1 | 452 / 600*^(6)^* | 1 - 15 | 128 - 144 | 0 / 12 / 5 |
| 1ANU | c2A | SD1 | 228 / 600*^(6)^* | 1 - 12 | 125 - 138 | 0 / 9 / 2 |
| 1UBQ | ubiquitin | SS | 227 / 600*^(6)^* | 1 - 8 | 63 - 76 | 0 / 5 / 2 |
| 1TIT | I27 | S | 217 / 600*^(6)^* | 1 - 15 | 77 - 89 | 0 / 3 / 4 |
| 2QMT | GB1 |  | 184/ 400[[7](#_ENREF_7)] | 1 - 10 | 49 - 56 | 0 / 7 / 1 |
| 1HZ6 | Protein L | SS | 151 / 600*^(6)^* | 2 - 13 | 55 - 64 | 0 / 8 / 2 |
| 1PGA | Protein G | SS | 190 / 600*^(6)^* | 1 - 10 | 49 - 56 | 0 / 6 / 2 |
| 1OWW | ^1^FNIII (fibronectin) | SD2 | 224 / 600*^(6)^* | 1 - 15 | 80 - 93 | 0 / 1 / 2 |

# Section E. Matlab script to select H-bonds between terminal strands.

%Input file should be an excel file with H-bonds in the protein, determined

%by Discovery studio visualizer.

function select_Hbonds_V3(filename)

data = readtable(filename);

start_N = 1; %first amino acid of N-terminal Beta-strand

end_N = 10; %last amino acid of N-terminal Beta-strand

start_C = 55; %first amino acid of C-terminal Beta-strand

end_C = 62; %last amino acid of C-terminal Beta-strand

result = [];

for i = 1: size(data,1) % Analyze for each H-bond whether it is located between N-and C-terminal strands

f = data{i,1};

f = f{1,1};

parts = strsplit(f, ':');

distance = data{i,9};

acceptor = parts{1,2};

accept_aa = acceptor(isstrprop(acceptor,'digit'));

accept_aa = str2num(accept_aa);

donor = parts{1,4};

donor_aa = donor(isstrprop(donor,'digit'));

donor_aa = str2num(donor_aa);

if start_N <= accept_aa & accept_aa <= end_N & start_C <= donor_aa & donor_aa <= end_C & distance <= 3.2

Index = i;

index = table(Index);

Hbond = [data(i,:), index];

result = [result; Hbond];

elseif start_C <= accept_aa & accept_aa <= end_C & start_N <= donor_aa & donor_aa <= end_N & distance <= 3.2

Index = i;

index = table(Index);

Hbond = [data(i,:), index];

result = [result; Hbond];

end

end

%Write H-bonds between N-and C-terminal strands to excel file.

writetable(result, strcat(filename,'_selection.xls'));

end

# Section F. Reduced thermal stability of *Mp*AFP RII tetra-tandemer in absence of calcium.

Differential scanning calorimetry (DSC) shows a higher thermal stability of *Mp*AFP RII tetra-tandemer observed in the presence of 10 mM calcium compared to the absence of calcium. In presence of 10 mM calcium the melting temperature of RII was 62.9 °C, while in absence of calcium the melting temperature was 38.9 °C (Figure S7). Measurements of *Mp*AFP RII tetra-tandemer in the presence of calcium were performed on ~1 mg/ml RII tetra-tandemer, in 50 mM Tris-HCl (pH 9), 200 mM NaCl, 10 mM CaCl_2._ For measurements in the absence of calcium, 15 mM EDTA was added to the buffer.


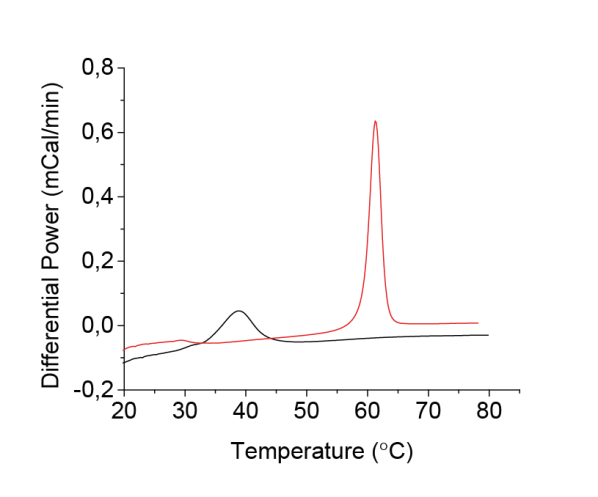


**Figure H.** *Differential scanning calorimetry curves of MpAFP RII tetra-tandemer in presence of more than 100 equivalents Ca^2+^ (red, T_m_ = 62,9 °C) and less than 1 equivalent Ca^2+^ (black, T_m_ = 38,9 °C).*

# Section G. GFP unfolding statistics.

The unfolding of GFP in *Mp*AFP RII8-GFP was analyzed in 20 force curves having five or more *Mp*AFP RII unfolding peaks. Since GFP is sandwiched in the middle of eight *Mp*AFP RII repeats and has a low mechanical stability compared to *Mp*AFP RII, it is very likely to be unfolded in these force experiments. However, in only three force curves (15%) was an GFP unfolding peak obtained (Fig S9A), with an average unfolding force *F*_u_ = 88 ± 7 pN and unfolding length *∆L* = 74.4 ± 3.9 nm. In other force curves, the GFP unfolding peak was either absent (50%, Fig S14B) or masked by non-specific interactions between the AFM tip and the substrate (35%, Fig S14C).


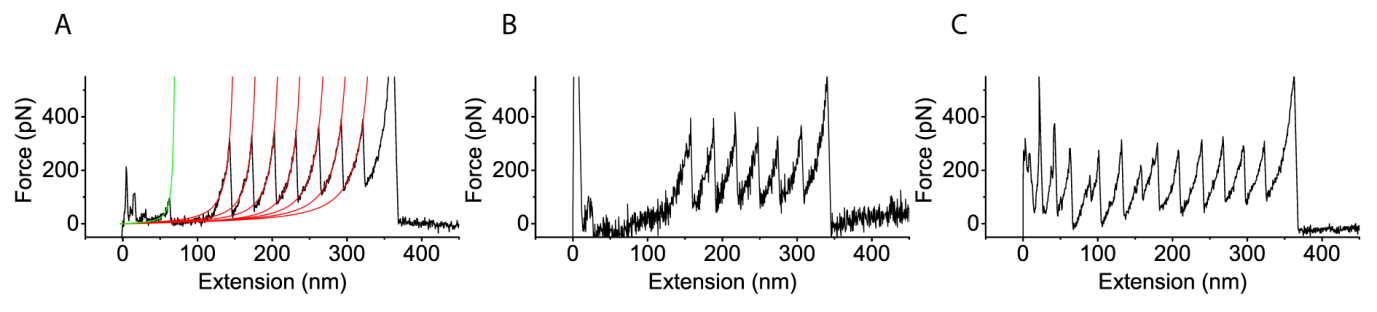


**Figure I.** *Example curves of MpAFP RII8-GFP with five or more MpAFP RII unfolding peaks, showing a GFP unfolding peak (A), absence of GFP unfolding peak (B), or masking of the GFP unfolding peak due to non-specific interactions between the AFM tip and the substrate (C). In A, a worm-like chain (WLC) model fit of the GFP peak is shown in green, and WLC fits of the MpAFP RII peaks are shown in red.*

# Section H. *Mp*AFP RII_8_-GFP absorbance and fluorescence spectra

Since GFP unfolding peaks were absent in some force curves with five or more RII unfolding peaks of *Mp*AFP RII_8_-GFP (see section S7), absorbance and fluorescence spectra of the protein were recorded (Figure S15). The extinction coefficients of the *Mp*AFP RII_8_-GFP protein are ε_280_ = 77810 M^-1^cm^-1^ at 280 nm [[8](#_ENREF_8)] and ε_489_ = 55000 M^-1^cm^-1^ at 489 nm.[[9](#_ENREF_9)] From the absorption spectra (Fig. S10A) we derive c = 0.10 mM based on A_280_ absorbance, and c = 0.09 mM based on A_489_ absorbance. The recorded fluorescence spectrum shows a maximum at 515 nm (Figure S15B) which is slightly shifted compared to the reported value of 508 nm.[[9](#_ENREF_9)] The spectra imply that the GFP protein is overall correctly folded within the RII repeats in the construct. However, incorrect folding might occur among single molecules in force spectroscopy experiments.

**
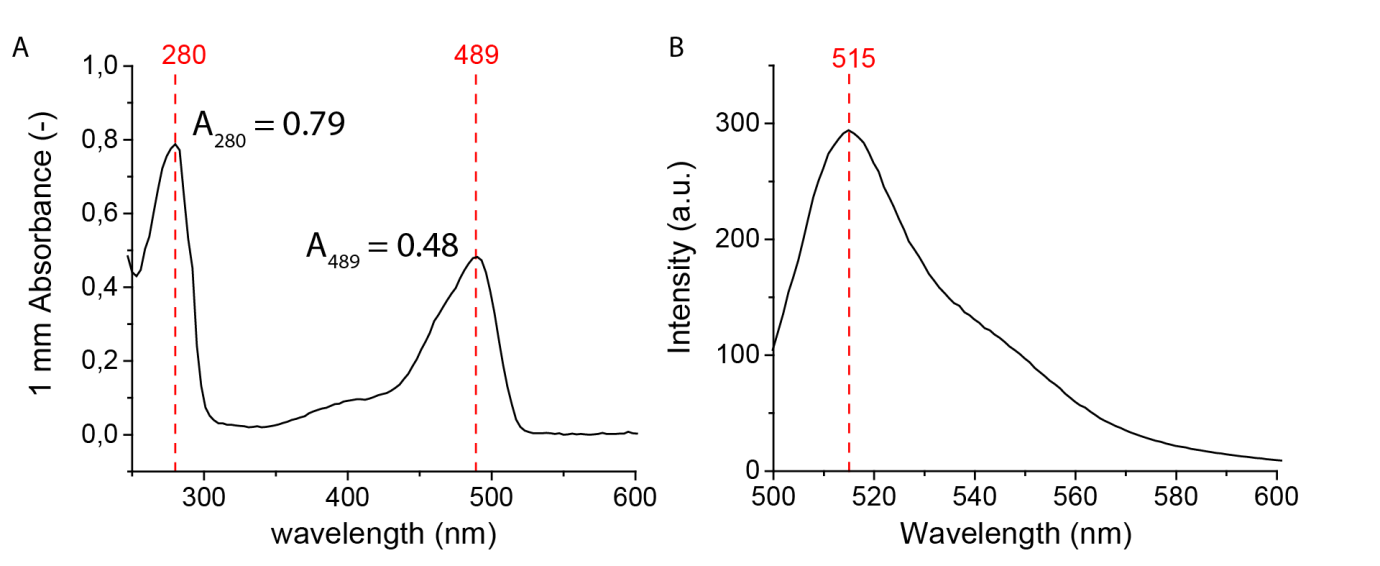
**

**Figure J.** *Absorbance (A) and fluorescence (B) spectra of MpAFP RII_8_-GFP. Absorption values at 280 and 489 nm are indicated.*

# Section I. Dynamic light scattering on *Mp*AFP RII_8_-GFP and *Mh*Lap RII_8_

Dynamic light scattering experiments were performed on 1 mg/ml aqueous solutions of *Mp*AFP RII_8_ and *Mh*Lap RII_8_ (50 mM Tris-HCl (pH 9), 200 mM NaCl, 10 mM CaCl_2_) to check whether the polyproteins are well soluble or instead aggregate in the presence of calcium. The solutions contained 0.5 mM of the reducing agents TCEP (*Mh*Lap RII_8_) or DTT (*Mp*AFP RII_8_-GFP) to reduce the two C-terminal cysteines and were filtered over a 0.2 µm filter (Whatman) before the dynamic light scattering (DLS) experiments (Figure S16). The (near-)monomodal correlation functions and corresponding size distributions centered around *D*_H_ ≤ 14 nm reveal that the octamers are well soluble (i.e., do no aggregate) under these conditions in the presence of calcium.


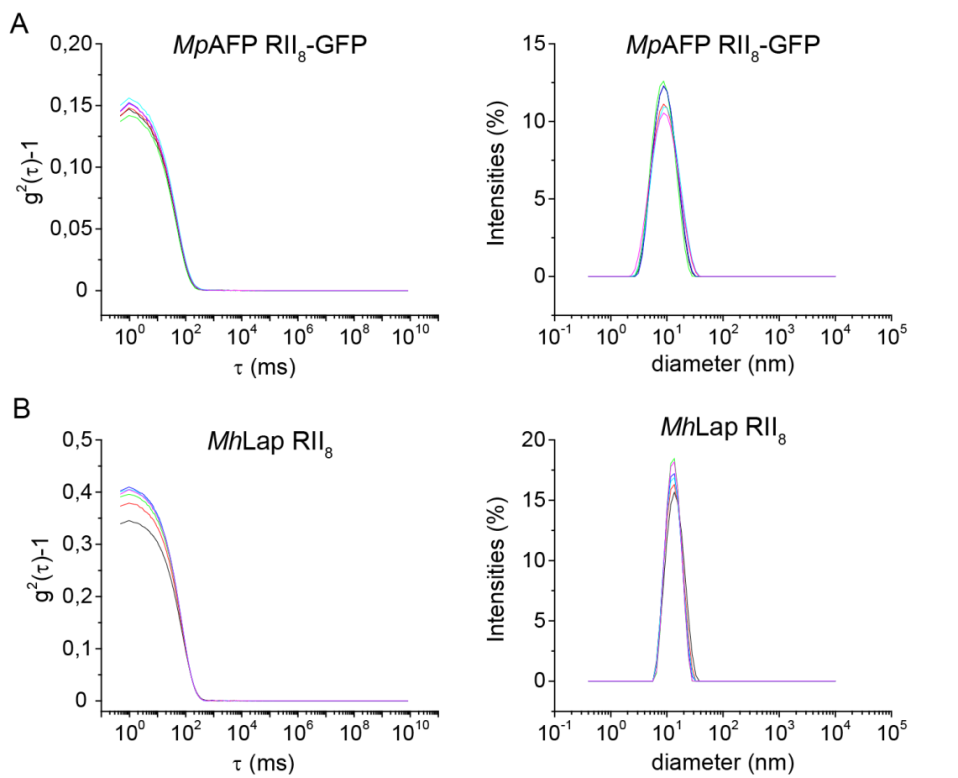


**Figure K.** *Results of dynamic light scattering experiments on MpAFP RII_8_-GFP (A) and Mhlap RII_8_ (B). The (near-)monomodal correlation functions and corresponding size distributions (centered around a hydrodynamic radius R_H_ = 4 ± 1 nm for MpAFP RII_8_-GFP and R_H_ = 7 ± 1 nm for MhLap RII_8_) reveal that the octamers do no aggregate under these conditions in the presence of calcium.*

**SUPPORTING REFERENCES**

1. Carrion-Vazquez M, Oberhauser AF, Fowler SB, Marszalek PE, Broedel SE, et al. (1999) Mechanical and chemical unfolding of a single protein: a comparison. Proceedings of the National Academy of Sciences 96: 3694-3699.

2. Rounsevell R, Forman JR, Clarke J (2004) Atomic force microscopy: mechanical unfolding of proteins. Methods 34: 100-111.

3. Kelley LA, Mezulis S, Yates CM, Wass MN, Sternberg MJ (2015) The Phyre2 web portal for protein modeling, prediction and analysis. Nature protocols 10: 845-858.

4. <http://www.sbg.bio.ic.ac.uk/phyre2/>.

5. <http://evans.rc.fas.harvard.edu/pdf/smnr_2009_Kwan_Eugene.pdf>.

6. Hoffmann T, Tych KM, Hughes ML, Brockwell DJ, Dougan L (2013) Towards design principles for determining the mechanical stability of proteins. Physical Chemistry Chemical Physics 15: 15767-15780.

7. Hoffmann T, Dougan L (2012) Single molecule force spectroscopy using polyproteins. Chemical Society Reviews 41: 4781-4796.

8. <http://web.expasy.org/protparam/>.

9. Patterson G, Day RN, Piston D (2001) Fluorescent protein spectra. Journal of cell science 114: 837-838.
